# Acute kidney injury following fatty liver ischemia-reperfusion injury: indirect protection by hepatic ferroptosis inhibition

**DOI:** 10.3389/fphys.2025.1672201

**Published:** 2025-11-19

**Authors:** Wenjun Zhang, Zachary Rokop, Shan Li, Christina R. Ferreria, Shunhua Guo, Weinian Shou, Pierre Dagher, Chandan K. Sen, Sylvester Black, Chandrashekhar Kubal

**Affiliations:** 1 Department of Surgery, Indiana University School of Medicine, Indianapolis, IN, United States; 2 Department of Pediatrics, Wells Center for Pediatric Research, Indiana University School of Medicine, Indianapolis, IN, United States; 3 Bindley Bioscience Center, Purdue University, West Lafayette, IN, United States; 4 Department of Pathology and Laboratory Medicine, Indiana University School of Medicine, Indianapolis, IN, United States; 5 Department of Medicine, Indiana University School of Medicine, Indianapolis, IN, United States; 6 Department of Surgery, McGowan Institute for Regenerative Medicine, University of Pittsburgh School of Medicine, Pittsburgh, PA, United States; 7 Department of Surgery, The Ohio State University School of Medicine, Columbus, OH, United States

**Keywords:** acute kidney injury, fatty liver, ischemia-reperfusion injury, ferroptosis, apoptosis, inflammation

## Abstract

**Background:**

The association between hepatic ischemia-reperfusion injury (hIRI) in steatotic livers and subsequent acute kidney injury (AKI) is well established. Ferroptosis plays a critical role in fatty liver IRI. However, whether ferroptosis also contributes to secondary AKI following hIRI remains unclear.

**Methods:**

hIRI was induced in mice fed either a high-fat, high-sucrose diet (HFD) or a normal diet (ND) to mimic the AKI commonly observed clinically after fatty liver transplantation. Kidney injury mechanisms were evaluated using histopathology, RNA sequencing, electron microscopy, and biochemical assays. Ferroptosis in the kidney was assessed by quantifying ACSL4, 4-hydroxynonenal (4-HNE), and AA-PE in homogenates and tissue sections. In parallel experiments, the lipid peroxidation inhibitor Liproxstatin-1 (Lip-1) was administered prior to hIRI to inhibit ferroptosis.

**Results:**

AKI severity was markedly increased in HFD-fed mice compared to ND controls following hIRI. Histological, transcriptomic, and cytokine analyses revealed that apoptosis and inflammation were the primary mechanisms of kidney injury after HFD + hIRI. Kidney levels of ACSL4 and 4-HNE were not significantly elevated in either group after hIRI. Lip-1 treatment significantly reduced both liver injury and AKI in HFD-fed mice but showed no protective effect in ND-fed animals.

**Conclusion:**

Apoptosis and inflammation are the prominent kidney injury mechanisms involved in AKI following fatty liver IRI. Although ferroptosis may not be directly involved in the renal injury, anti-ferroptotic intervention mitigates AKI, supporting the concept that ferroptosis-mediated liver injury may serve as the primary upstream trigger in this context.

## Introduction

Acute kidney injury (AKI) is a common complication, particularly in critically ill patients, and is associated with increased morbidity and mortality (Hoste et al., 2018). Dysfunction of other organ systems, such as cardiovascular and hepatic systems, is often a major contributor to AKI (Lee et al., 2018). The link between hepatic IRI (hIRI), and subsequent AKI is well established (Saidi et al., 2014). Consistent with this, AKI occurs frequently following liver transplantation (LT) with reported incidence ranging from 20% to 60% (Hussaini et al., 2019; Durand et al., 2018). Clinical evidence suggested that even mild or transient post-LT AKI has been associated with prolonged hospital stays and increased morbidity and mortality (Hilmi et al., 2008).

Compared to lean livers, fatty livers are more susceptible to IRI, as demonstrated in various experimental models (Tevar et al., 2011; Wan et al., 2008; Rokop et al., 2024) and in clinical settings (Selzner and Clavien, 2001; McCormack et al., 2011). Fatty liver disease is also linked to a higher risk of AKI. During liver transplantation, steatotic grafts are particularly vulnerable to IRI and subsequent AKI (Jadlowiec et al., 2020; Kalisvaart et al., 2019). For example, post-LT AKI was observed in 52.5% of patients receiving moderately steatotic grafts compared to only 16.8% in recipients of non-steatotic livers (Jadlowiec et al., 2020). Despite the recognized association between fatty liver IRI and AKI, the underlying mechanisms remain poorly defined. It has been suggested that the release of vasoactive substances—such as reactive oxygen species, proinflammatory cytokines, and chemokines—from the injured liver contributes to the pathogenesis of AKI in this context (Umbro et al., 2016; Klune and Tsung, 2010). Previously, we demonstrated enhanced ferroptosis in fatty livers subjected to IRI, as evidenced by increased lipid oxidation and elevated ferroptosis markers such as 4-hydroxynonenal (4-HNE) and ACSL4 (Doll et al., 2017; Zhang et al., 2023). Treatment with the ferroptosis inhibitor Liproxstatin (Lip-1) for 7 days prior to IRI significantly attenuated liver injury sustained in HFD-fed mice (Rokop et al., 2024). Given that ferroptosis also plays a significant role in AKI resulting from kidney IRI or in rsponse to cisplatin-induced nephrotoxicity (Li et al., 2024; Song et al., 2024), it remains unclear whether ferroptosis contribute to AKI through a liver-kidney signaling axis under the setting.

In this study, using a previously established murine fatty liver IRI model in our laboratory (Rokop et al., 2024), we aimed to investigate the primary mechanisms of AKI following fatty liver IRI and explored the potential involvements of ferroptosis in AKI in this process.

## Methods

### Animal care and high fat diet treatment

All experiments were conducted using C57BL6 male mice, obtained from Envigo (Indianapolis, IN). All animal care and experimental procedures were approved by the Indiana University Institutional Use and Care Committee (IACUC) and were performed in accordance with the guidelines. Mice were housed in sterile, individual ventilated cages under a 12-h light/dark cycle with *ad libitum* access to food and water. Hepatic steatosis was induced by feeding 10-week-old mice with a high fat, high sucrose diet (HFD; 12.6% protein, 29% fat, 46.2% carbohydrates; TD 160785, Envigo) for 12 weeks (Rokop et al., 2024; Ishimoto et al., 2013). Age-matched control mice were maintained on a normal chow diet (ND; 20% protein, 10% fat, 70% carbohydrates) for the same duration. At the end of HFD or ND treatment, mice underwent either hIRI or sham surgery.

### Liver IRI and tissue collection

**FIGURE 1:**
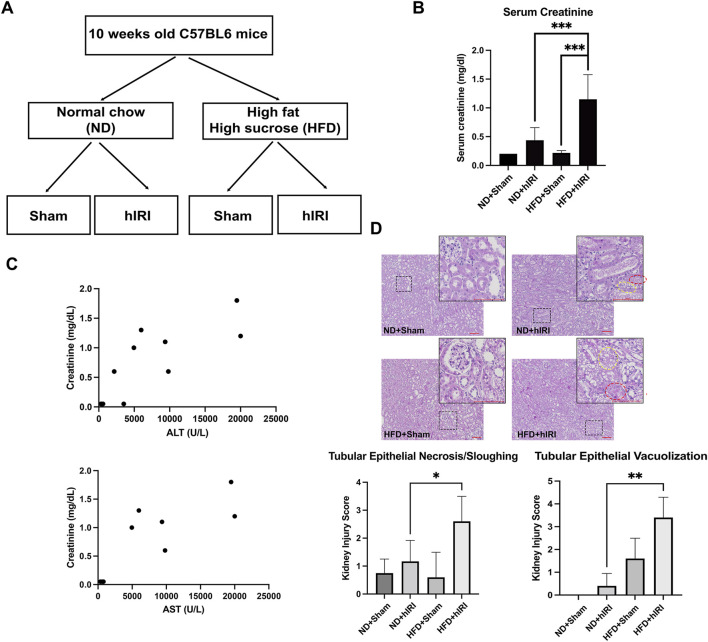
Exacerbated AKI in fatty liver IRI mouse model. **(A)** Schematic illustration of the experimental design of the liver IRI animal model in control and fatty liver mice. **(B)** Serum Creatinine in ND + Sham, ND + hIRI, HFD + Sham, and HFD + hIRI mice. **(C)** Correlation analysis of serum ALT or AST with serum creatinine in HFD + hIRI animal group*, n =* 9. *P <* 0.01, *R =* 0.8023 and *0.8103* respectively. **(D)** H&E staining demonstrated an increase intubular epithelial sloughing and vacuolization in both HFD + hIRI as compared to ND fed mice and HFD + Sham mice. Scale bar: 100 μm. Upper panel: pathological quantification of tubular epithelial sloughing and vacuolization in mouse kidney section from animal groups as indicated. *p < 0.05, **p < 0.01, ***p < 0.001. *n = 8* for each animal group. Red circles indicated the tissue area with tubular epithelial vacuolization, and yellow circle indicated the tissue area with tubular epithelial sloughing.

After 12 weeks of dietary intervention (ND or HFD), mice were randomly assigned to undergo either sham or hIRI surgical procedure. The experimental groups were designated as ND + sham, ND + hIRI, HFD + sham, and HFD + hIRI, respectively (Figure 1A). The hIRI and sham procedures were performed as previously described (Rokop et al., 2024). Briefly, mice were deeply anesthetized with 3.5% isoflurane via inhalation, and a midline abdominal incision was made to expose the liver and the hepatic pedicle (containing hepatic artery, portal vein, and bile duct). The segment of hepatic pedicle supplying the left lateral and right median liver lobes was occluded using a non-traumatic micro-aneurysm clamp for 60 min (Devey L et al., 2008). Ischemia of the targeted lobes was confirmed by visualization of liver blanching and laser doppler (LDF). After 60 min, the clamp was removed to allow reperfusion via restoration of blood flow. The body temperature was measured throughout the procedure and maintained between 36 °C and 37 °C. Following reperfusion, the fascial and skin layers were closed, and animals were allowed to recover from anesthesia. After 24 h, the animals were anesthetized with 5% isoflurane via inhalation for retro-orbital blood collection, followed by euthanasia via cervical dislocation for tissue harvesting. For sham procedures, laparotomy was performed, and the same protocol was followed except for the pedicle clamping.

### Serum analysis for kidney function

Biochemical quantification of kidney injury was performed by measurement of serum creatinine levels using the Piccolo Xpress chemistry analyzer.

### Histological analysis

Mouse kidneys were fixed with 4% paraformaldehyde. Paraffin embedding, sectioning, and hematoxylin & eosin staining were performed following standard protocols. Histological analysis was performed by a trained renal pathologist and scored as previously reported (Lee et al., 2024; Ban et al., 2022). Histologic evaluation of the kidney injury was performed on the H&E stained tissue sections. The kidney injury was mainly manifested in the tubules which demonstrated nuclear and cytoplasmic debris in the tubular lumens or sloughing of the epithelial cells into the lumens, and cytoplasmic vacuolization of the tubular epithelial cells. Tubular injury was scored based on the percentage of injured area as follows: 0, no damage; 1, injured area 1%–10%; 2, injured area 11%–25%; 3, injured area 26%–50%; and 4, injured area 50% or more. The evaluation was performed together, blinded to any group of diet or treatment.

### Immunohistochemistry and immunofluorescence

Immunohistochemical (IHC) staining was performed on formalin-fixed, paraffin-embedded (FFPE) mouse kidney sections using kits from Vector Laboratories (Newark, CA), following the manufacturer’s instructions. The primary antibodies used for IHC included anti-cleaved Caspase-3 (Cell Signaling Technology, MA), anti-F4/80, Ly6B.2 (Novus Biologicals, CO), and anti-ACSL4 (Santa Cruz Biotechnology, TX). Immunofluorescence (IF) staining was performed on frozen kidney tissue sections. Nuclear counterstaining was carried out using Hoechst 33,343 (ThermoFisher Scientific, MA). For IF, anti-4-HNE (Life Technologies, CA) and anti-cleaved Caspase-3 (Cell Signaling Technology, MA) were used as primary antibodies. Images were captured using the Leica DMi8 microscope equipped with the Leica DFC7000 T imaging system. Quantification of fluorescent signal was performed by first scanning and acquiring the images of the entire tissue sections using the ZEISS AxioScan 7 slide scanner. Ten representative regions of interest (ROI), each measuring an area of 10,000 um^2^, were selected for each slide. The fluorescence intensity ratio of primary antibody signal to Hoechst nuclear signal was measured. Mean fluorescent intensity was determined by averaging values across all ROIs from each sample.

### Transmission electron microscopy (TEM)

Transmission electron microscopy (TEM) was performed to assess ultrastructural kidney damage in ND + hIRI and HFD + hIRI animal groups at the Indiana University School of Medicine Center for Electron Microscopy (ICEM). Briefly, freshly harvested kidneys were immediately minced and fixed in 3% glutaraldehyde prepared in 0.1 M phosphate buffer. The tissues were then processed, embedded, and sectioned into ultrathin slices (80–90 nm thick). Sections were examined using a Tecnai™ Spirit TEM (Thermo Fisher, Hillsboro, OR), and digital images were captured using a charge-coupled device (CCD) camera (Advanced Microscopy Techniques, Danvers, MA).

### Multiplex for cytokine level in kidney lysate

Kidney tissues were flash-frozen in liquid nitrogen and subsequently pulverized under liquid nitrogen using a mortar and pestle. Approximately 10 mg of tissue from each sample was lysed in RIPA buffer (Santa Cruz Biotechnology) and further diluted in phosphate-buffered saline (PBS). Cytokine profiling was performed using a multiplex assay conducted by Eve Technologies (Calgary, Canada).

### ELISA for ACSL4

To determine ACSL4 levels in mouse kidney tissue, tissue was mechanically ground into a fine powder under liquid nitrogen with mortar and pestle, and then further homogenized in RIPA buffer on ice using a TissueLyser (Retsch). The homogenates were centrifuged at 12,000 × g for 10 min at 4 °C, and the resulting supernatants were collected for analysis. ACSL4 concentrations were measured using the MyBioSource ACSL4 ELISA kit (MBS9324691) according to the manufacturer’s instructions. Results are expressed as nanograms of ACSL4 protein per milligram of tissue.

### AA-PE mass spectrometry

Mass spectrometric analysis of kidney tissue extracts was performed to detect arachidonic acid–containing phosphatidylethanolamine [PE (18:0/20:4)] using ultra-performance liquid chromatography coupled with high-resolution mass spectrometry (UPLC-HRMS). Separation was achieved on a Waters BEH C18 column (1.7 μm, 2.1 × 100 mm). Mass spectrometric analysis was conducted on an Agilent 6545 Q-TOF mass spectrometer operated with Agilent MassHunter Acquisition software (v. B.06). The identity of PE (18:0/20:4) was confirmed by tandem mass spectrometry (MS/MS) using database matching and comparison with an analytical standard (Cayman Chemical, #61216-62-4).

### RNA sequencing analysis

The mice from ND + sham, ND + hIRI, HFD + sham, and HFD + hIRI (n = 3 each group) animal groups were subjected to the same feeding and surgical procedure as we described above for transcriptomic analysis. To capture early molecular events induced by hIRI, the reperfusion period was shortened to 6 h before kidney tissue collection. Total RNA was extracted using TRIzol® reagent (Thermo Science) according to manufacturer’s instruction. Paired-end cDNA library preparation and sequencing were carried out following the standard protocol at the Center of Medical Genomics of Indiana University School of Medicine. Sequence reads were mapped to the reference genome using STAR (Spliced Transcripts Alignment to a Reference (Dobin et al., 2013). RNA-seq data quality was assessed by calculating the distribution of reads across different genomic features (exons, introns, splice junctions, intergenic regions, promoters, UTRs, etc.) using bamUtils (Breese and Liu, 2013). Differential gene expression analysis was performed with edgeR, as described previously (Robinson et al., 2010). Gene Ontology set enrichment analysis (GSEA) was performed using cluster Profiler’s gseGO function from the clusterProfiler package on the list of differentially expressed genes identified by edgeR (Yu et al., 2012).

### Ferroptosis inhibition

To inhibit ferroptosis, mice fed either ND or HFD for 12 weeks were administered with the potent ferroptosis inhibitor Lip-1 (MedChemExpress, NJ) at a dose of 10 mg/kg via intraperitoneal injection once daily for 7 consecutive days. An additional dose of Lip-1 (10 mg/kg) was administered 1 hour prior to hIRI surgery. In the vehicle control group, mice received the same volume of 0.1% DMSO at the corresponding time points. Mice then underwent either hIRI or sham surgery as previously described. After 24 h, animals were euthanized, and serum and kidney tissues were collected for further analysis.”

### Statistical analysis

All values are presented as mean ± standard deviation. Statistical significance was determined by 1-way ANOVA using GraphPad Prism (GraphPad Software). For all figures, “*” represents statistical significance at the following levels: * <0.05, ** <0.01, *** <0.001, **** <0.0001.

## Results

### Exacerbated AKI in fatty liver IRI mouse model

We previously established a hIRI model in HFD-fed mice and successfully replicated the severe liver injury pattern often observed in clinical settings during liver transplantation involving steatotic grafts (Rokop et al., 2024). Serum alanine transaminase (ALT) and aspartate aminotransferase (AST), measured 24 h after hIRI or sham surgeries, were significantly elevated in mice from HFD + IRI group as compared to their ND fed control counterparts (ND + IRI), confirming the induction of more severe liver injury developed in fatty liver model (Rokop et al., 2024). To determine whether HFD + hIRI is also associated with acute kidney injury (AKI), we measured serum creatinine levels across experimental groups following either sham surgery or hIRI (occlusion of blood flow to the left and median right lobes for 1 h) with 24 h of reperfusion. Serum creatinine levels were significantly elevated in HFD + hIRI animals compared to ND + hIRI mice (1.08 ± 0.42 vs. 0.47 ± 0.18; P < 0.0001) (Figure 1A), indicating worsened AKI in the context of fatty liver. Furthermore, serum creatinine levels in HFD + hIRI mice strongly correlated with ALT and AST elevations (P < 0.01, R = 0.8023 and 0.8103, respectively) (Figure 1B), suggesting that kidney injury severity correlates hepatic injury in these animals. Histological analysis revealed that kidneys from HFD + sham animals already exhibited tubular epithelial vacuolization compared to ND + sham controls (1.6 ± 0.9 vs. 0 ± 0), indicating baseline kidney pathology from HFD feeding. hIRI significantly worsened renal injury in HFD-fed mice, as evidenced by increased tubular epithelial sloughing and cell death (Kidney Injury Score: 2.4 ± 1.1 vs. 1.0 ± 0.84; P = 0.03) and enhanced vacuolization (3.4 ± 0.9 vs. 0.4 ± 0.5; P < 0.001) compared to ND + hIRI animals (Figure 1C).

### Transcriptomic profiling of injury-associated signalingpathways in kidneys of HFD-fed mice following hIRI

**FIGURE 2:**
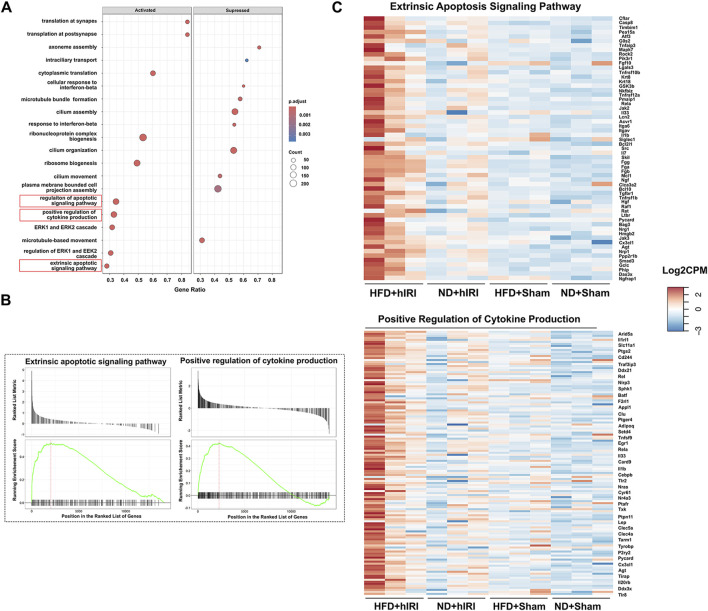
Transcriptomic analysis of kidneys following hIRI or sham procedure in lean and fatty mouse model. **(A)** Dot plot showing the top activated and suppressed signaling pathway enriched by GO BP analysis when the kidney transcriptomics from HFD + hIRI animal group was compared to that from ND mice after hIRI. **(B)** Enrichment score plot showing the activated and suppressed gene number associated with extrinsic apoptosis pathway and positive regulation of cytokine production pathway enriched by GO BP analysis when the kidney transcriptomics from HFD + hIRI animal group was compared to that from ND mice after hIRI. **(C)** Heatmap showing the top upregulated genes associated with extrinsic apoptosis pathway and positive regulation of cytokine production in the kidney tissue from HFD+hIRI animal group as compared to that from HFD+Sham, ND+hIRI and ND+Sham. *n = 3* for each animal groups.

To investigate signaling mechanisms contributing to renal injury in the setting of fatty liver IRI, we performed RNA-seq analysis on kidney and liver homogenates collected 6 h after hIRI or sham surgical procedures from all experimental groups, as in prior experiments. Gene Ontology Biological Process (BP) analysis was conducted to identify enriched gene sets involved in the biological pathways associated with acute kidney injury (AKI). Following hIRI, there was a significant upregulation of gene sets associated with the regulation of apoptotic signaling, the extrinsic apoptotic pathway, and cytokine production in HFD-fed animals as compared to ND-fed mice (Figures 2A–C). KEGG pathway analysis further revealed increased activation of apoptosis, IL-17 signaling, and the complement and coagulation cascades in the kidneys of HFD + hIRI mice relative to ND + hIRI and their respective sham controls (Supplementary Figure S1A–D). These results suggest that hIRI induces heightened inflammation and apoptosis in the kidneys of HFD-fed mice. Comparison of BP terms between HFD sham and ND sham kidneys revealed increased enrichment of pathways related to leukocyte chemotaxis and migration, granulocyte recruitment, and cytokine production. In contrast, pathways associated with cilium assembly and organization were downregulated in HFD sham kidneys (Supplementary Figure S2). These findings indicate that a proinflammatory microenvironment may already be present in kidneys of HFD-fed mice, consistent with the tubular epithelial vacuolization observed histologically in the HFD + sham group. Furthermore, Gene Ontology Biological Process (GO:BP) analysis of liver RNA-seq data showed significant enrichment of gene sets related to the positive regulation of interleukin-1 (IL-1) production, interleukin-6 (IL-6) production, and tumor necrosis factor (TNF) superfamily signaling in steatotic livers after IRI compared with lean livers subjected to the same procedure (Supplementary Figure SA–B3). Consistently, KEGG pathway analysis indicated increased enrichment of the chemokine signaling pathway and reduced enrichment of peroxisome-related pathways in HFD + hIRI versus ND + hIRI livers (Supplementary Figure S3C–D). While these results are based on a murine model, they suggest early hepatic signaling cascades that priming toward AKI in the context of metabolic stress.

### Enhanced renal apoptosis in HFD-fed mice following hIRI

**FIGURE 3:**
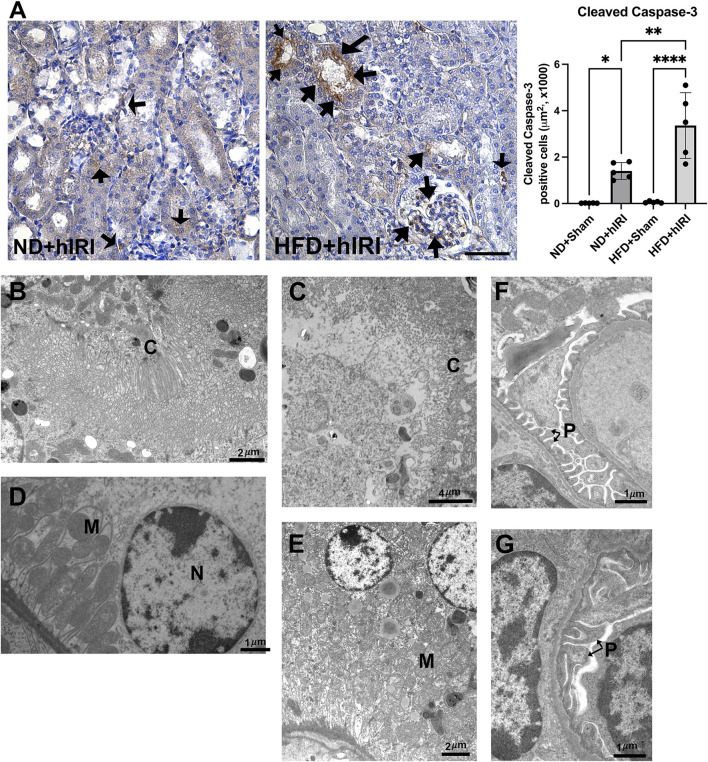
Renal apoptosis following fatty liver IRI. **(A)** Left, Representative IHC staining images of cleaved Caspase-3 on kidney sections from the indicated animal groups. Scale bar: 100 μm. Right, quantification of cleaved Caspase-3 positive cells per μm^2^. Arrows indicated apoptotic cells in the kidney sections. n = 5 for each animal group, *p < 0.05, **p < 0.01. **(B–G)** TEM images showing the ultrastructure of kidney from ND + hIRI **(B–F)** and HDF + hIRI **(C–G)** animal groups. **(C)** Cilia, M: Mitochodria, N: Nucleus. Glomerulus in mouse from ND + hIRI **(F)** showing pedicles (P) of podocytes and tight space of the filtration slits (arrow) (Scale bar is as indicated in each panel). In animal kidneys from HFD + hIRI group **(G)**, the glomerulus showing the fusion of pedicles (P) (arrow).

Given that RNA-seq analysis of kidney at 6 h post-hIRI in HFD-fed mice revealed upregulation of gene sets associated with apoptotic signaling pathways, including the extrinsic apoptotic pathway, we performed immunohistochemical (IHC) staining for cleaved caspase-3—a well-established marker of apoptosis—on kidney sections collected 24 h after hIRI. Compared to ND + hIRI animals, HFD + hIRI kidneys showed markedly increased cleaved caspase-3 staining, indicating enhanced apoptosis (Figure 3A). Apoptotic cells were observed in both tubular and extra-tubular compartments, including within glomerular capillaries. Transmission electron microscopy (TEM) further confirmed tubular injury, revealing brush border disruption and sloughing in the proximal tubules of HFD + hIRI animals (Figure 3C), in contrast to relatively preserved structures in ND + hIRI kidneys (Figure 3B). Additional cytoplasmic abnormalities—including mitochondrial cristae damage, vacuolization, and irregular contours—were evident in tubular epithelial cells (Figures 3D,E). Moreover, glomerular ultrastructure in ND + hIRI kidneys displayed intact podocyte pedicles with narrow filtration slits (Figure 3F), while HFD + hIRI kidneys exhibited fused pedicles and swollen morphology, along with widened filtration slit spaces (Figure 3G). Collectively, these observations confirm that hepatic IRI in the context of fatty liver promotes activation of apoptotic pathways and leads to increased apoptosis in both tubular epithelial and glomerular cells, potentially contributing to the exacerbated AKI phenotype observed in HFD-fed animals.

### Fatty liver IRI triggers inflammatory response in kidney

**FIGURE 4:**
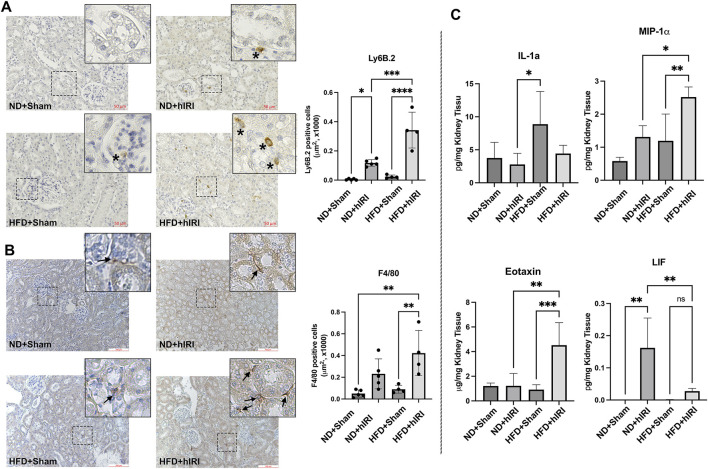
Inflammation in kidneys following hIRI. **(A)** Left, Representative IHC staining images Ly6B.2 on kidney sections from the indicated animal groups. Scale bar: 50 μm. Right, quantification of infiltrated Ly6B.2 positive neutrophil cells per mm^2^. **(B)** Representative IHC staining images of F4/80 on kidney sections from the indicated animal groups. Scale bar: 100 μm. Right, quantification of infiltrated F4/80 positive macrophage per μm^2^. **(C)** Multiplex assay for inflammatory cytokine in kidney from the indicated animal groups. *p < 0.05, **p < 0.01, ***p < 0.001. *n = 5* for each animal group.

Transcriptomic analysis revealed an upregulation of the gene sets associated with inflammation and cytokine production in kidneys from HFD + hIRI mice compared to ND + hIRI animals. To validate these findings, we performed IHC staining for Ly6B.2 and F4/80, the well-established markers for neutrophil and macrophage, on kidney tissue sections. As expected, HFD + hIRI kidneys exhibited significantly increased infiltration of neutrophils, inflammatory monocytes, and activated macrophages compared to ND + hIRI and sham controls (Figures 4A,B). Multiplex cytokine analysis of kidney homogenates further confirmed elevated levels of inflammatory mediators, including IL-1α, Eotaxin, and MIP-1α, in HFD + hIRI animals (Figure 4C), indicating robust renal inflammation. Interestingly, while the anti-inflammatory cytokine leukemia inhibitory factor (LIF) was significantly upregulated in ND + hIRI kidneys compared to their sham controls, hIRI failed to induce LIF expression in the kidneys of HFD-fed animals. This observation suggests an altered response to hepatic IRI in the setting of fatty liver, which may further contribute to the heightened renal inflammation and injury observed in these animals.

### Contribution of ferroptosis in renal injury after fatty liver hIRI

**FIGURE 5:**
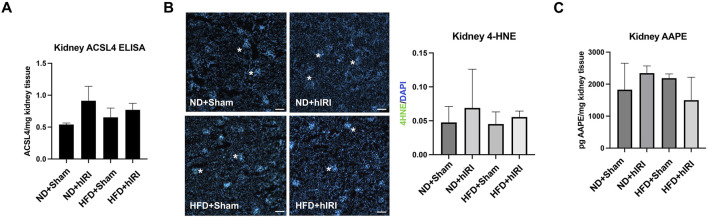
Contribution of renal ferroptosis in AKI following fatty liver IRI. **(A)** ELISA assay showing ACSL4 level are not augmented in HFD + IRI or ND + IRI mouse kidney as compared to their Sham controls. **(B)** kidneys from hepatic IRI in fatty mouse model showing no increase of 4-HNE staining signal (in green) in either HFD + IRI or ND + IRI kidney as compared to their Sham controls. Nuclei were counterstained with Hoechst 33,343. Scale bar: 25 μm. **(C)** Mass Spectrometric Analysis demonstrated that kidney AA-PE level was not elevated in HFD + IRI or ND + IRI mouse kidney as compared to their Sham controls. *n = 5* for each animal group.

**FIGURE 6:**
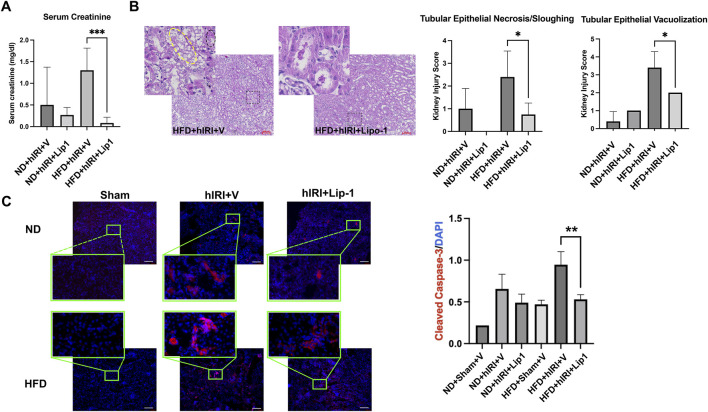
Administration of ferroptosis inhibitor, Lip-1, mitigates AKI following fatty liver IRI. **(A)** Serum creatinine level in ND- and HFD-fed mice in 24 h post hIRI. The mice were pre-treated with liproxstatin-1 (Lip-1) or control vehicle for 7 days. **(B)** H&E staining demonstrated Lip-1 treatment ameliorate tubular epithelial sloughing and vacuolization in kidneys of HFD + IRI mice as compared to control vehicle treated animals. Right panel: pathological quantification of tubular epithelial sloughing and vacuolization in mouse kidney section as indicated. Scale bar: 100 μm *n = 5* for each animal group. *p < 0.05. **(C)** Left, Representative immunofluorescence staining images of cleaved Caspase-3 (in red) on the kidney sections from the indicated animal groups. Nuclei were counterstained with Hoechst 33,343. Scale bar: 100 μm. Right, quantification of cleaved Caspase-3 positive cells per μm^2^. *n = 5* for each animal group. **p < 0.01.

We previously reported that treatment with Liproxstatin-1 (Lip-1), a potent ferroptosis inhibitor (Zilka O et al., 2017), significantly reduced 4-HNE levels as well as serum ALT and AST levels in HFD-fed mice subjected to hIRI, compared to vehicle-treated controls. These findings supported a crucial role for ferroptosis in mediating hepatic injury during IRI in fatty livers (Rokop et al., 2024). Interestingly, while RNA-seq analysis indicated there was a significant elevation of *ACSL4*, (Doll et al., 2017), in kidneys from HFD-fed animals 6 h post-hIRI (Supplementary Table S2; Supplementary Figure S4); ELISA measurements of ACSL4 protein levels in kidneys 24 h post-hIRI showed no significant differences between groups (Figure 5A). To further assess the involvement of renal ferroptosis in AKI associated with fatty liver IRI, we evaluated lipid peroxidation in the kidney by immunofluorescence staining of 4-HNE. Notably, 4-HNE levels were not elevated in either HFD + hIRI or ND + hIRI kidneys relative to sham controls (ND or HFD + Sham) at 24 h post-hIRI (Figure 5B). Arachidonic acid–phosphatidylethanolamine (AA-PE), a polyunsaturated fatty acid, is highly susceptible to oxidation and serves as a critical lipid substrate for ferroptosis execution (Kagan et al., 2017). Mass spectrometric quantification of (AA-PE) in kidney tissue revealed no significant difference between sham and hIRI groups under either dietary condition (Figure 5C). These results suggest that ferroptosis is unlikely to be directly involved in the pathogenesis of AKI following liver IRI in HFD-fed mice. Given our previous findings that Lip-1 attenuates hepatic injury in fatty liver IRI (Rokop et al., 2024), we investigated whether this protective effect extends to kidneys. Lip-1 was administered intraperitoneally (10 mg/kg daily) for 7 days prior to hIRI, as was performed in our prior study (Rokop et al., 2024). Remarkably, Lip-1 treatment led to a significant reduction in serum creatinine levels in HFD + hIRI mice compared to vehicle-treated controls (Figure 6A). Histological analysis of H&E-stained kidney sections revealed reduced tubular epithelial necrosis and vacuolization in Lip-1–treated HFD + hIRI mice (Figure 6B). Furthermore, cleaved caspase-3 staining showed attenuated apoptosis in the kidneys of Lip-1–treated HFD + hIRI mice compared to ND + hIRI and HFD + sham controls (Figure 6C). Collectively, these findings suggest that while ferroptosis may not directly contribute to the exacerbated AKI observed in fatty liver IRI, anti-ferroptotic intervention with Lip-1 nonetheless ameliorates renal injury—likely through indirect effects mediated by protection of the liver.

## Discussion

This study investigated the mechanisms underlying AKI in a setting of fatty liver IRI. While prior studies have explored the AKI mechanisms following liver IRI (Lee et al., 2009; Park et al., 2010), few have specifically addressed it in the context of fatty liver. Clinically, despite technical advances in liver transplantation, AKI remains a common complication, increasing morbidity and mortality. In liver transplantation setting, hIRI occurs during the procurement, cold storage, and surgical procedures (Hussaini et al., 2019; Durand et al., 2018). The liver-kidney axis is well documented in conditions such as hepatorenal syndrome and cirrhosis-associated chronic kidney disease (Adebayo and Wong, 2023; Appenrodt and Lammert, 2018). Our findings confirm and extend our and others prior work by showing that fatty liver IRI results in more severe AKI than lean liver IRI, suggesting that steatotic livers exacerbate systemic injury to remote organs—including the kidney—via inflammation and oxidative stress.

We previously demonstrated that fatty livers are more susceptible to ischemic injury (Rokop et al., 2024). However, the specific mechanisms linking hepatic injury to remote organ damage such as AKI remain poorly defined. Comprehensive understanding of the molecular pathways contributing to AKI in this context is critical for the development of diagnostic biomarkers and therapeutic strategies. Previous reports have shown that inflammation plays a central role in AKI across multiple contexts, including sepsis- or ischemia-associated AKI (Zarbock et al., 2023; Lee and Jang, 2022). In line with these findings, we found fatty liver IRI induced substantial renal inflammation, characterized by increased infiltration of neutrophils, inflammatory monocytes, and activated macrophages, along with elevated levels of pro-inflammatory cytokines such as IL-1α, Eotaxin, and MIP-1α. Previous reports have established that IL-1α can act as a proinflammatory signal for kidney damage (Anders, 2016), where as Eotaxin and MIP-1α (CCL3) are potent chemokines that contribute to the recruitment of immune cells and promote AKI (Wada et al., 1999; Badenska et al., 2025; Chen et al., 2020). Interestingly, the level of leukemia inhibitory factor (LIF) was markedly upregulated in kidney from ND + hIRI mice, but not in those from HFD + hIRI mice. Previous studies have shown that LIF acts as a protective factor against ischemic acute kidney injury (Hu et al., 2024) and is involved in renal epithelial regeneration following AKI (Yoshino et al., 2003). This impaired LIF response may reflect a diminished renal reparative capacity under conditions of systemic inflammation, contributing to worsened AKI in fatty liver IRI. Consistent with this, RNA sequencing analysis revealed a significant upregulation of inflammation-related gene sets in kidneys from HFD + hIRI mice relative to ND + hIRI controls, further confirmed the systemic inflammation and inflammatory “spillover” from fatty liver contribute to AKI.

Apoptosis is another established mechanism contributes to overall renal tubular cell death IRI-induced AKI (Lee et al., 2009; Linkermann. et al., 2014a). In our model, cleaved caspase-3 staining demonstrated marked increase of apoptosis in kidneys from HFD + hIRI animals. TEM analysis confirmed structural damage, including brush border disruption and sloughing in proximal tubules of both ND + hIRI and HFD + hIRI mice. However, additional ultrastructural abnormalities—such as cytoplasmic vacuolization and endothelial contour irregularities—were noted only in HFD + hIRI animals, particularly in peritubular capillary regions. These coordinated transcriptional changes are spanning from receptors (*Tnfrsf1b*, *Tnfrsf10b*, *Tnfrsf12a*, *Ltbr*), initiator caspase (*Casp8*), adaptors (*Pycard*), downstream effectors (*Pmaip1*), to regulatory feedback nodes (*Cflar*, *Tnfaip3*, *Bcl2l1*, *Mcl1*) are consistent with activation of the death-receptor/caspase-8 axis upstream of the cleaved caspase-3 signal that resulted in exacerbated renal apoptosis and AKI in HFD + hIRI animals. These findings underscore the severity of tubular and vascular injury under steatosis conditions. Necrosis is well-recognized form of cell death in renal IRI (Fleig et al., 1987). Previously, we used Evans Blue (EBD) to assess plasma-membrane leakage, a feature commonly associated with necrosis, and confirmed a marked increase of necrosis in fatty livers subjected to IRI (Rokop et al., 2024). However, using the same methods, we did not detect significant necrosis in the kidneys of either HFD + IRI or ND + IRI mice (data not shown), suggesting that necrosis is not a major contributor to fatty liver IRI-associated AKI.

Our data show that fatty liver IRI produces more severe AKI than lean liver IRI, with apoptosis and inflammation as the predominant injury mechanisms. These results support a model in which hIRI in steatotic livers aggravates remote organ injury—including the kidney—through circulating inflammatory mediators, activation of extrinsic apoptotic pathways, and oxidative stress. Consistent with this, our prior work demonstrated higher levels of pro-inflammatory cytokines (IL-1α, IL-6, IL-12p40, IFN-β1, IL-17, and TNF-α) in HFD + hIRI liver tissue compared with ND + hIRI, indicating a more intense inflammatory milieu in fatty livers after IRI (Rokop et al., 2024). Likewise, RNA-seq revealed enrichment of gene sets related to positive regulation of interleukin-1 and interleukin-6 production and TNF superfamily signaling in HFD + hIRI livers, along with increased chemokine-signaling pathways and decreased peroxisome pathways relative to ND + hIRI. Because peroxisome-related genes are essential for very-long-chain fatty-acid catabolism, plasmalogen synthesis, and detoxification of hydrogen peroxide, their suppression would be expected to reduce antioxidant capacity and heighten susceptibility to oxidative injury (Fransen et al., 2012). Together, these findings implicate liver-derived pro-inflammatory cytokines (IL-1, IL-6, TNF-α), extrinsic apoptosis signaling (TNF-α/TNFSF), and metabolic oxidative stress (e.g., ROS) as circulating drivers that initiate and amplify AKI in the context of fatty liver IRI.

Ferroptosis has recently been implicated in the pathogenesis of various acute and chronic liver disorders (Chen et al., 2022; Capelletti et al., 2020). Recent evidence shows that a high-fat diet (HFD) reshapes the gut microbiota—reducing diversity, depleting barrier-supportive microbial environment, enriching bile-tolerant pathobionts via taurine-conjugated bile acids, and diminishing short-chain fatty acid (SCFA) production (Hu et al., 2025; Devkota et al., 2012). This HFD-induced dysbiosis, through portal LPS/TLR4 priming and disrupted bile-acid/SCFA signaling, can lower hepatic lipid-peroxidation defenses (e.g., the ACSL4/LPCAT3/GPX4 axis) and sensitize steatotic livers to ferroptosis during ischemia–reperfusion injury (Yao and Li, 2023). Furthermore, steatotic livers are typically deficient in reduced glutathione (GSH), a key antioxidant that neutralizes reactive oxygen species (ROS). This deficiency increases vulnerability to ROS accumulation and predisposes the tissue to ferroptosis-driven injury (Junzhe et al., 2024). Although ferroptosis has previously been implicated in the development of AKI of renal IRI and direct nephrotoxic injury (Linkermann et al., 2014b; Zhao Z et al., 2020), our analyses revealed no significant elevation of canonical ferroptosis markers—including 4-HNE, ACSL4 protein, or AA-PE—in kidneys at 24 h post-hIRI. These findings argue against ferroptosis as a direct renal mechanism in this model and instead support a remote injury paradigm, distinct from models of direct renal insult. Nevertheless, the renoprotective effects of Lip-1 in fatty liver IRI suggest that inhibition of hepatic ferroptosis mitigates downstream kidney injury. Furthermore, despite Lip-1 being a potent and specific ferroptosis inhibitor, off-target effects—such as modulation of oxidative stress or survival pathways—cannot be excluded. Future studies using alternative radical-trapping antioxidants (Scarpellini et al., 2023), GPX4 activators to reduces lipid hydroperoxides (Qi et al., 2020), and complementary interventions targeting upstream components of extrinsic apoptosis (e.g., caspase-8 activation, FADD recruitment, or receptor/ligand blockade) are warranted to confirm our findings.

Of note, RNA-seq revealed early (6-h) upregulation of ACSL4 and other antioxidant defense genes in the kidneys of HFD + hIRI mice, suggesting a potential priming of ferroptotic pathways during the early phase of injury. These findings warrant further time-course studies to clarify the temporal dynamics of ferroptosis-related signaling in the kidney during fatty liver IRI. Furthermore, the future in-depth definitive dissection of the liver-to-kidney signaling axis (e.g., specific cytokines/lipid mediators, receptor–ligand pairs, and cell-type sources) will provide a more detailed mechanistic insights on how hepatic ferroptosis impact renal injury under the setting of fatty liver IRI.

In conclusion, our study provides new insights into the pathophysiology of AKI following fatty liver IRI. Inflammation and apoptosis are key mediators of renal injury. While ferroptosis does not appear to be directly involved in the kidney, targeting ferroptosis in the liver offers a promising strategy to reduce remote organ injury. These findings advance our understanding of the liver-kidney axis and highlight ferroptosis inhibition as a potential therapeutic avenue for preventing AKI in the setting of fatty liver transplantation or surgery.

## Data Availability

The original contributions presented in the study are publicly available. This data can be found here: https://www.ncbi.nlm.nih.gov/geo/query/acc.cgi?acc=GSE309904.

## Supplementary material

The Supplementary Material for this article can be found online at: https://www.frontiersin.org/articles/10.3389/fphys.2025.1672201/full#supplementary-material

SUPPLEMENTARY FIGURE S1
**(A)** Dot plot showing the top activated and suppressed signaling pathway enriched by GO KEGG analysis of the kidney transcriptomics from HFD+hIRI and HFD sham versus ND+hIRI and ND sham. **(B-D)**, Heatmap showing the top upregulated genes associated with apoptosis pathway **(B)**, IL-17 signaling pathway **(C)**, and complement and co-agulation cascades **(D)** in the kidney tissue from HFD+hIRI animal group as compared to HFD+Sham, ND+hIRI and ND+Sham mice.

SUPPLEMENTARY FIGURE S2
**(A)** Dot plot showing the top activated and suppressed signaling pathway enriched by GO BP analysis of HFD+sham versus ND+ sham.

SUPPLEMENTARY FIGURE S3
**(A,B)** Dot plot and Ridgeline plot showing the top activated and suppressed signaling pathway enriched by GO BP analysis of the liver transcriptomics from HFD+hIRI versus ND+hIRI mice. **(C,D)** Dot plot and Ridgeline plot showing the top activated and suppressed signaling pathway enriched by GO KEGG analysis of the liver transcriptomics from HFD+hIRI versus ND+hIRI mice.

SUPPLEMENTARY FIGURE S4 Heatmap showing the expression of the genes associated with ferroptosis that enriched by GO BP analysis of the kidney transcriptomics from different animal groups.
