# Supplementary material for: Acute kidney injury following fatty liver ischemia-reperfusion injury: indirect protection by hepatic ferroptosis inhibition
Source: Front Physiol. 2025 Nov 19;16:1672201. doi: 10.3389/fphys.2025.1672201 (PMC12673840; doi:10.3389/fphys.2025.1672201)
Supplement: Supplementary file 1 [file DataSheet1.pdf]

Supplemental Material:

Supplemental tables:

Supplemental table 1. Differential expression analysis of kidney transcriptomes identified genes significantly up- or downregulated in HFD+hIRI mice compared to ND+hIRI controls. A false discovery rate (FDR) of <0.05 was used as the threshold for significance.

Supplemental table 2. The list of the genes associated with ferroptosis weret enriched by GO BP analysis of the kidney transcriptomics from HFD+hIRI mice as compared to ND+hIRI controls.

Supplemental Figure 1

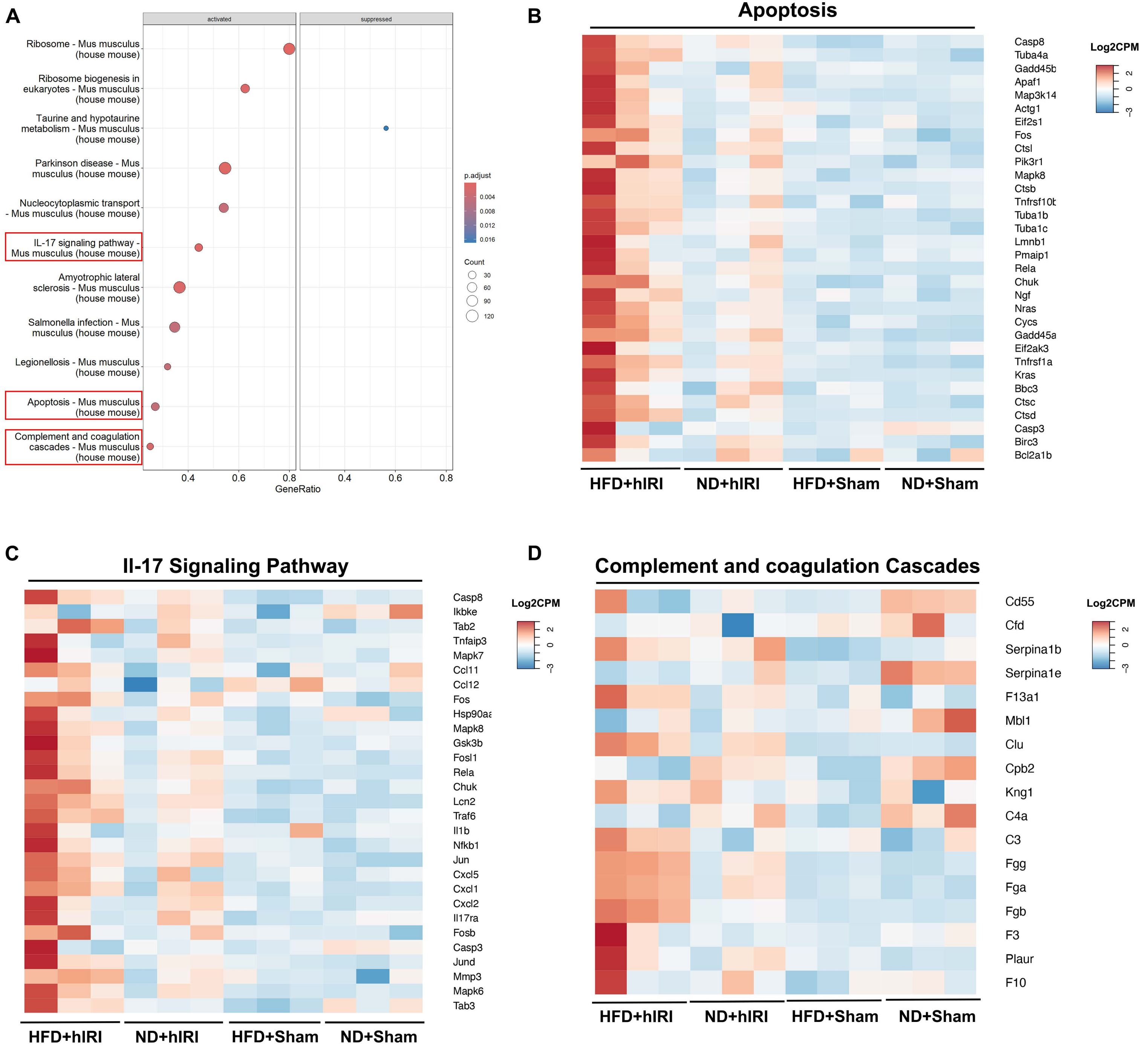

Supplemental Figure 2

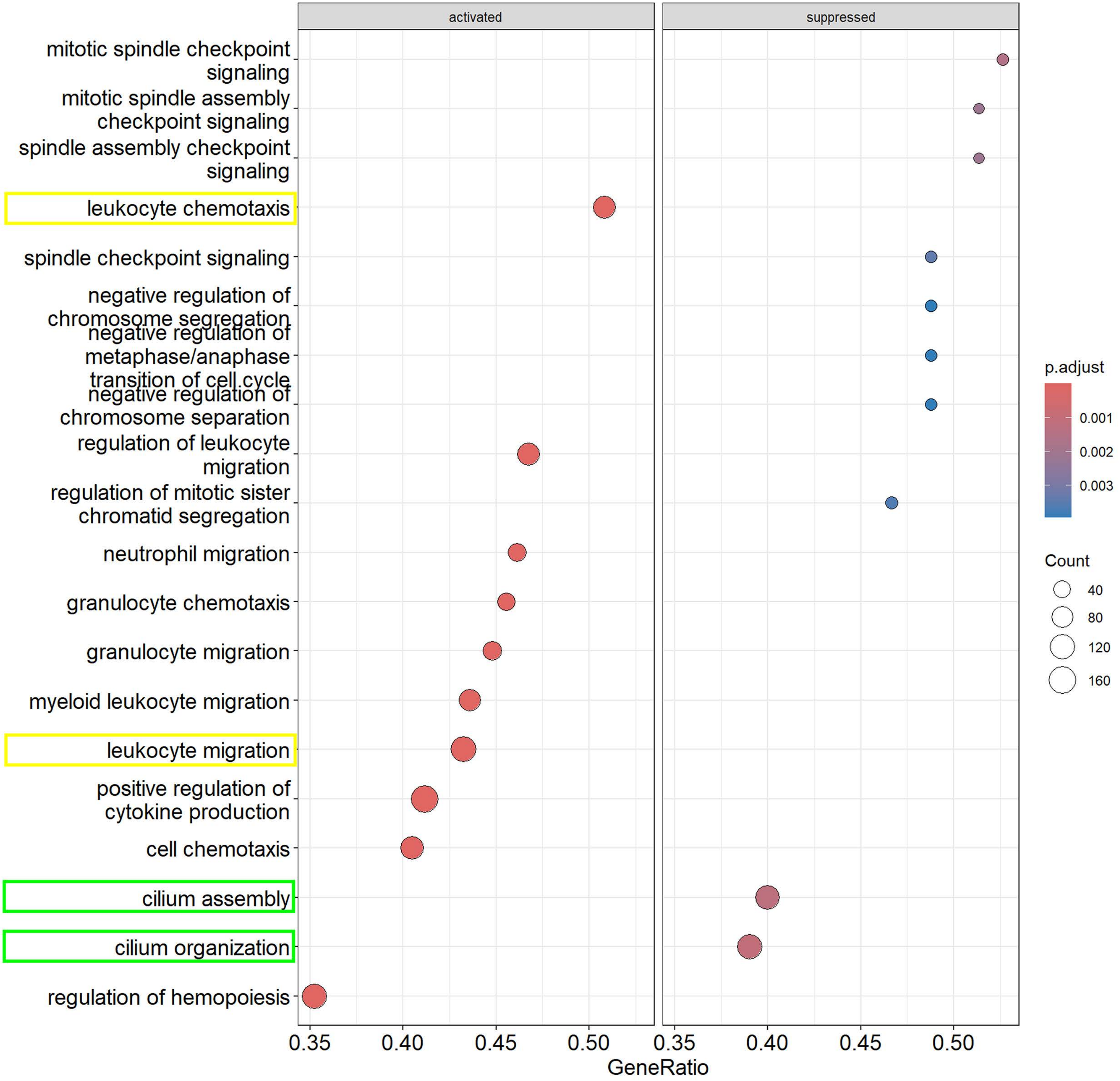

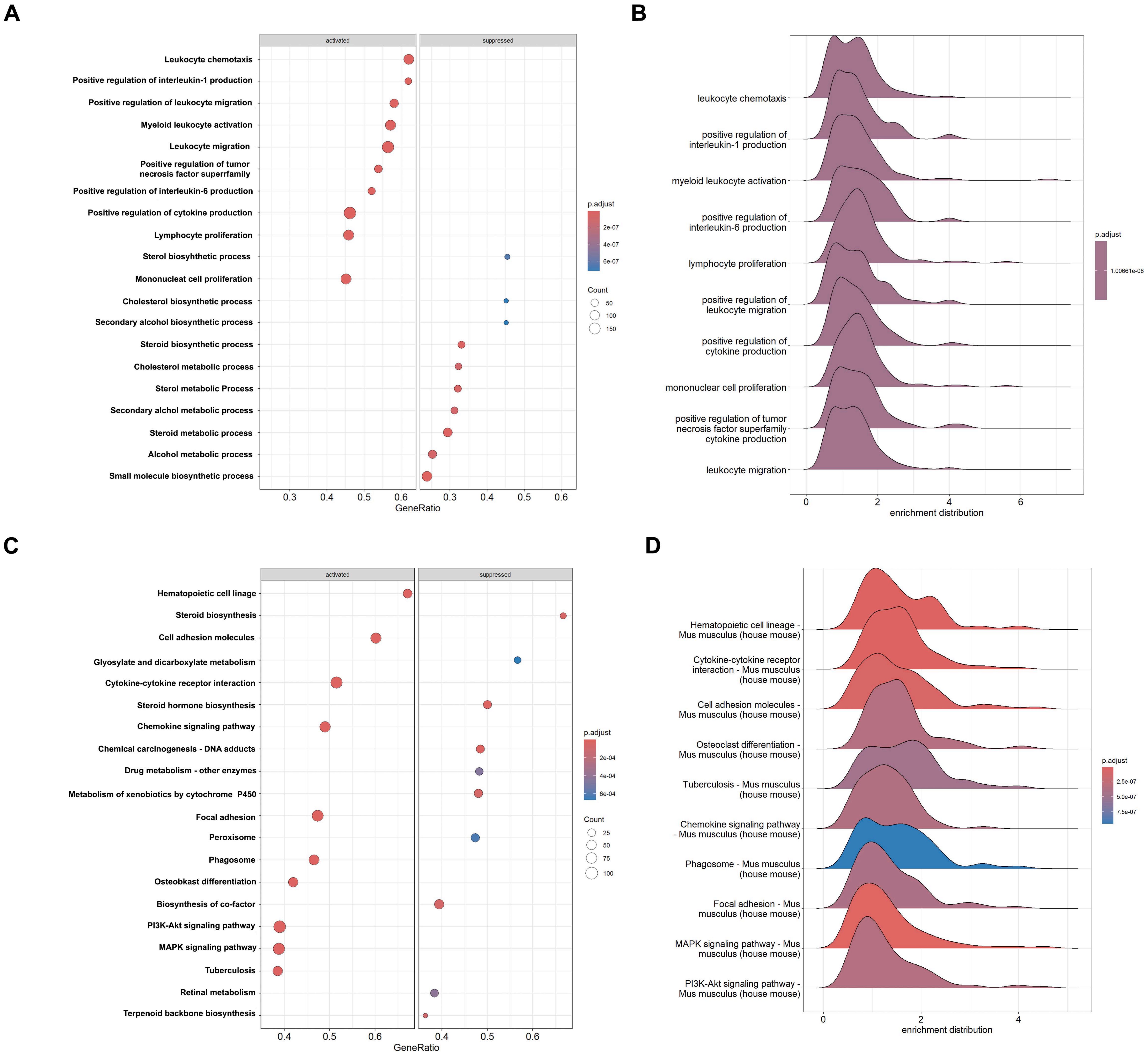
A
activated
suppressed
Leukocyte chemotaxis
Positive regulation of interleukin-1 production
Positive regulation of leukocyte migration
Myeloid leukocyte activation
Leukocyte migration
Positive regulation of tumor necrosis factor superrfamily
Positive regulation of interleukin-6 production
Positive regulation of cytokine production
Lymphocyte proliferation
Sterol biosynthetic process
Mononucleat cell proliferation
Cholesterol biosynthetic process
Secondary alcohol biosynthetic process
Steroid biosynthetic process
Cholesterol metabolic process
Sterol metabolic Process
Secondary alchol metabolic process
Steroid metabolic process
Alcohol metabolic process
Small molecule biosynthetic process
0.3 0.4 0.5 0.6 0.3 0.4 0.5 0.6
GeneRatio
p.adjust
2e-07
4e-07
6e-07
Count
50
100
150
B
leukocyte chemotaxis
positive regulation of interleukin-1 production
myeloid leukocyte activation
positive regulation of interleukin-6 production
lymphocyte proliferation
positive regulation of leukocyte migration
positive regulation of cytokine production
mononuclear cell proliferation
positive regulation of tumor necrosis factor superfamily cytokine production
leukocyte migration
0 2 4 6
enrichment distribution
p.adjust
1.00661e-08
C
activated
suppressed
Hematopoietic cell lineage
Steroid biosynthesis
Cell adhesion molecules
Glyosylate and dicarboxylate metabolism
Cytokine-cytokine receptor interaction
Steroid hormone biosynthesis
Chemokine signaling pathway
Chemical carcinogenesis - DNA adducts
Drug metabolism - other enzymes
Metabolism of xenobiotics by cytochrome P450
Focal adhesion
Peroxisome
Phagosome
Osteobkast differentiation
Biosynthesis of co-factor
PI3K-Akt signaling pathway
MAPK signaling pathway
Tuberculosis
Retinal metabolism
Terpenoid backbone biosynthesis
0.4 0.5 0.6 0.4 0.5 0.6
GeneRatio
p.adjust
2e-04
4e-04
6e-04
Count
25
50
75
100
D
Hematopoietic cell lineage - Mus musculus (house mouse)
Cytokine-cytokine receptor interaction - Mus musculus (house mouse)
Cell adhesion molecules - Mus musculus (house mouse)
Osteoclast differentiation - Mus musculus (house mouse)
Tuberculosis - Mus musculus (house mouse)
Chemokine signaling pathway - Mus musculus (house mouse)
Phagosome - Mus musculus (house mouse)
Focal adhesion - Mus musculus (house mouse)
MAPK signaling pathway - Mus musculus (house mouse)
PI3K-Akt signaling pathway - Mus musculus (house mouse)
0 2 4
enrichment distribution
p.adjust
2.5e-07
5.0e-07
7.5e-07

Supplemental Figure 3

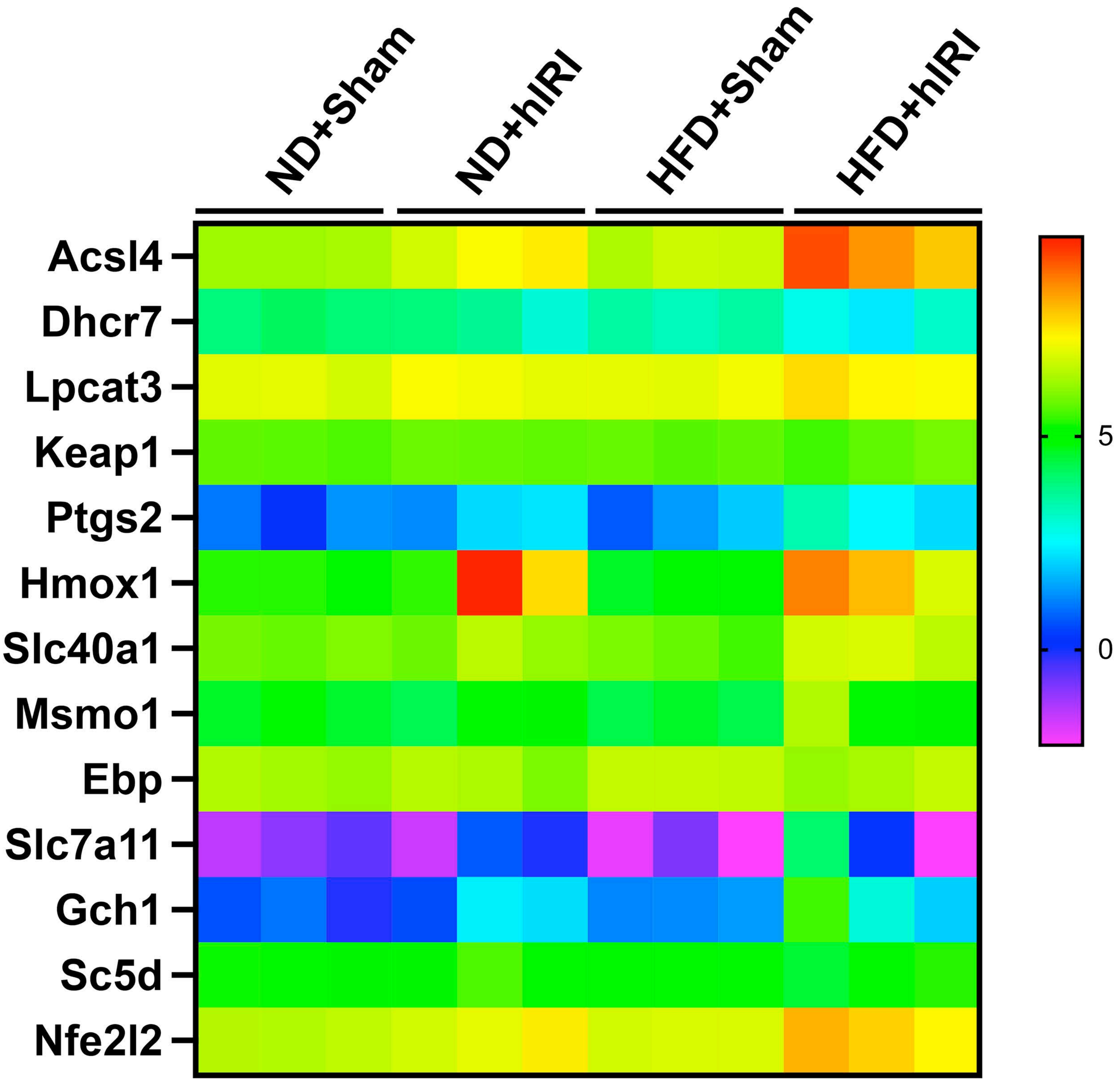

Gene Name	HFD_IRI_vs_ND_IRI_FC	HFD_IRI_vs_ND_IRI_logFC	HFD_IRI_vs_ND_IRI_PValue	HFD_IRI_vs_ND_IRI_FDR
Fgb	29.77844407	4.89619647	2.04594E-26	2.88294E-22
Vdr	-2.341844411	-1.227645228	3.39944E-20	2.39507E-16
Tmprss9	-6.853562463	-2.776854092	1.30398E-14	6.12481E-11
Kcng1	-28.42864714	-4.829273541	1.50811E-13	5.31269E-10
Acly	2.516158242	1.331222656	3.51661E-12	9.91051E-09
Fh1	2.448030396	1.291621471	1.6063E-11	3.77239E-08
Hyi	-1.880939425	-0.911453388	5.04085E-11	1.01472E-07
Mep1b	-2.519765488	-1.33328947	1.29632E-10	2.28331E-07
Igf2bp1	-5.964257471	-2.576342539	2.40852E-10	3.77094E-07
Gsto1	1.889979089	0.918370272	5.02005E-10	7.07375E-07
Map2k6	-2.787281613	-1.478858772	1.40098E-09	1.79465E-06
Abcd4	-2.30672359	-1.205845139	2.49249E-09	2.92681E-06
Gbp10	-14.68076539	-3.875855281	3.30544E-09	3.58284E-06
Hsd17b11	-1.925790292	-0.945450611	4.03594E-09	4.06217E-06
Apoe	2.163976993	1.113685161	4.87499E-09	4.57956E-06
Neil2	-4.168516661	-2.059534102	5.55657E-09	4.8936E-06
Slc16a1	2.506441383	1.325640495	6.4221E-09	5.32317E-06
Akr1d1	-1.852917653	-0.889798767	7.39965E-09	5.79269E-06
Mgst1	3.151049641	1.655832483	1.03312E-08	7.66195E-06
Trim7	-2.273791051	-1.185099684	1.29943E-08	9.15514E-06
Wfdc2	2.00312254	1.00225068	2.22673E-08	1.34318E-05
Tmigd1	-1.79251964	-0.841988926	2.23444E-08	1.34318E-05
Zmat1	-2.57794111	-1.366219308	2.28324E-08	1.34318E-05
Hdac5	-1.716806837	-0.779727727	2.28773E-08	1.34318E-05
Gsta2	2.427152469	1.279264739	3.0768E-08	1.73421E-05
Tubb2a	4.605872583	2.203474501	3.55704E-08	1.92778E-05
Ace	-2.357891799	-1.237497516	4.0613E-08	2.11955E-05
Hunk	-2.063292859	-1.044948609	4.2492E-08	2.13841E-05
Maob	2.444917174	1.289785592	5.16102E-08	2.50772E-05
Chst11	1.933928651	0.95153457	6.59656E-08	3.09841E-05
Lrg1	7.852444768	2.97314189	6.93153E-08	3.15072E-05
Fras1	-2.096743356	-1.068150285	8.17048E-08	3.59782E-05
Elovl2	2.101234824	1.0712374	8.72118E-08	3.72394E-05
Degs2	-2.582382054	-1.368702458	9.14044E-08	3.78818E-05
Fam25c	2.873761752	1.522940461	1.00648E-07	4.05209E-05
Arg2	3.43379994	1.779805987	1.16022E-07	4.54128E-05
Slc28a1	-2.031527972	-1.022565229	1.37017E-07	5.21811E-05
Fbxl2	-2.354327857	-1.23531524	1.49039E-07	5.506E-05
Myom3	-6.652524914	-2.733902008	1.52455E-07	5.506E-05
Srd5a2	-2.9350224	-1.553371514	1.56298E-07	5.506E-05
Acsm3	-6.053077511	-2.597668825	1.83954E-07	6.32219E-05
Fhod3	-2.318406489	-1.213133538	2.09502E-07	7.02879E-05
Plin2	7.081467602	2.824048383	2.27782E-07	7.38071E-05
Abcc3	2.301159537	1.202361008	2.30467E-07	7.38071E-05
Slc38a3	3.138962538	1.650287811	2.35873E-07	7.38598E-05
Dqx1	-2.408324012	-1.268029503	2.47333E-07	7.57645E-05
Slc35f3	-2.007132142	-1.005135602	2.94596E-07	8.83223E-05
Dscaml1	-9.000309018	-3.169974536	3.01434E-07	8.84896E-05
Cp	1.802694685	0.850155075	3.21097E-07	9.23385E-05
Tifa	3.074168785	1.620196377	3.51453E-07	9.90464E-05
Hs6st2	-3.416510105	-1.772523394	3.75564E-07	0.000103766
Neu1	-1.706970982	-0.771438533	5.01065E-07	0.000135759
Apob	1.973914239	0.98105931	5.10625E-07	0.000135759
Tmco3	-1.829871329	-0.871742206	6.03524E-07	0.000155103
Efcab12	-4.402141359	-2.138205473	6.14012E-07	0.000155103
Tfrc	3.995883601	1.998514558	6.16406E-07	0.000155103
Mas1	-8.61636661	-3.107079635	6.41665E-07	0.000158626
Zmynd10	-2.775729506	-1.472866985	6.56194E-07	0.000159421
Dnah11	-7.43017887	-2.893396942	6.68713E-07	0.000159709
4930481A15[illegible]	-2.541317373	-1.345576558	7.02574E-07	0.000164483
Nipal3	-2.049487522	-1.035263206	7.12047E-07	0.000164483
Erc2	-1.80852502	-0.854813557	8.2907E-07	0.000188426
Saa1	20.50531295	4.357925857	8.60574E-07	0.000190722
Gm4956	-3.462440739	-1.791789379	8.6624E-07	0.000190722
Immp2l	2.015129523	1.010872572	9.42336E-07	0.000204284
Lrrc56	-2.501237382	-1.322641984	1.0324E-06	0.000219422
Acsl4	2.632643596	1.396512224	1.0499E-06	0.000219422
Gm6300	-19.28278287	-4.26924137	1.05888E-06	0.000219422
Sox2ot	-5.232280452	-2.387439872	1.14097E-06	0.000231773
Acy1	-1.905106914	-0.929871963	1.15794E-06	0.000231773
BC024386	-2.622810366	-1.391113502	1.16783E-06	0.000231773
Gcnt4	2.05105077	1.036363203	1.23099E-06	0.000240915
Chrdl1	-3.66870676	-1.875271594	1.33349E-06	0.000257401
Bcas3	-1.651841635	-0.72407538	1.55924E-06	0.000296909

Tmem72	-2.069695832	-1.049418761	1.78189E-06	0.000334782
Armcx4	3.185893771	1.671698163	1.83914E-06	0.000340056
Nlrp6	-2.483791677	-1.312544175	1.85823E-06	0.000340056
Pnpla6	-1.608740354	-0.685931498	1.95543E-06	0.000353256
Ppp2r3a	-1.596662977	-0.675059821	2.01471E-06	0.000359357
Gm128	-3.768513356	-1.913995506	2.07445E-06	0.000365388
Osbp2	-1.677689819	-0.746476007	2.12007E-06	0.000367162
Coasy	-1.779593395	-0.831547649	2.13663E-06	0.000367162
Hrg	-2.30491107	-1.204711088	2.21017E-06	0.000375223
Ell3	-3.070836185	-1.618631553	2.24149E-06	0.000376009
Iqgap2	1.520762373	0.604794742	2.27116E-06	0.000376506
Papss1	-1.894567217	-0.921868326	2.34078E-06	0.000383534
Gm11992	-2.763360597	-1.466423835	2.38964E-06	0.000386443
Sox9	12.10919032	3.598030497	2.41338E-06	0.000386443
Serpina1c	-4.430999948	-2.14763231	2.45994E-06	0.000389472
Aqp1	-1.598521592	-0.676738231	2.65177E-06	0.000415178
Rpl39	1.708974827	0.773131146	2.76352E-06	0.000427921
Fgg	11.41043565	3.512281969	2.81838E-06	0.000431672
Alas2	2.054343471	1.03867741	3.10385E-06	0.000470284
Podn	-3.381746722	-1.757768612	3.20185E-06	0.000476938
Srd5a1	-2.044699357	-1.031888731	3.21546E-06	0.000476938
Plcd4	-4.280218538	-2.097684459	3.3134E-06	0.000481609
Cit	-3.312670689	-1.727994793	3.31531E-06	0.000481609
Plg	-13.16744822	-3.718903881	3.37903E-06	0.000485856
Cbr3	6.167250235	2.624627385	3.804E-06	0.000541436
Prkcz	-1.994772256	-0.996224043	4.11642E-06	0.000580044
Kcnj1	-1.946978725	-0.96123712	4.27126E-06	0.000595904
Ppp1r16a	-1.554006369	-0.635992416	5.07875E-06	0.000693502
Tfcp2	-2.105152796	-1.073924951	5.11436E-06	0.000693502
Prss8	-1.837668414	-0.877876473	5.12322E-06	0.000693502
Tnn	-3.204689923	-1.680184772	5.16768E-06	0.000693502
Cbs	-1.734716165	-0.794699628	5.35237E-06	0.000711511
Zfhx2	-2.09863261	-1.069449628	5.55391E-06	0.000728494
Akr1c14	-4.761507203	-2.251418315	5.58351E-06	0.000728494
Amdhd2	-1.517961337	-0.602135045	5.69986E-06	0.000736851
Slc22a28	-2.008120366	-1.005845746	5.81682E-06	0.000745134
Gad1	-7.506240641	-2.908090542	6.00727E-06	0.000762599
Slc5a6	-2.080061636	-1.056626279	6.11213E-06	0.000768983
Scrn2	-1.640651474	-0.714268798	6.35148E-06	0.000792023
Ypel3	-1.867157269	-0.900843449	6.65629E-06	0.000822753
C1qtnf3	-2.150659498	-1.104779129	6.74053E-06	0.000825919
Gc	5.2053716	2.380001158	7.57392E-06	0.00091658
Dzank1	-2.798533068	-1.484670795	7.61053E-06	0.00091658
Hfe	1.786286004	0.83696309	8.15748E-06	0.000974127
Bdh1	-2.056061952	-1.039883736	8.31332E-06	0.000984394
Mmd	2.16876126	1.116871248	8.61722E-06	0.001011877
Bbs9	-2.031311213	-1.022411289	8.82312E-06	0.001027493
Snx31	-3.250759838	-1.700776976	9.04061E-06	0.00104419
Serpinb6a	1.770721431	0.824337266	9.20603E-06	0.001054652
Bcat2	-1.571337205	-0.651992812	9.63069E-06	0.001089384
Smim10l2a	-2.698113405	-1.431950988	9.66383E-06	0.001089384
Fbf1	-1.739553205	-0.798716805	9.88421E-06	0.001097668
Tgfbr1	1.815810646	0.860613765	9.89311E-06	0.001097668
Kif20b	-4.282203365	-2.098353312	1.05743E-05	0.001164079
Slc16a6	6.123193426	2.614284257	1.09574E-05	0.001196904
Gsr	1.638000027	0.71193538	1.13408E-05	0.001229251
Gatsl3	-2.33903991	-1.225916478	1.16518E-05	0.001249368
Macrod2	-1.473449052	-0.559197177	1.17037E-05	0.001249368
Dbi	1.652258427	0.724439354	1.18074E-05	0.001250966
Dnajc12	-2.12119348	-1.084876219	1.20323E-05	0.001259574
Hsd3b2	-3.066148143	-1.616427403	1.21372E-05	0.001259574
Ifrd2	-1.902980853	-0.928261046	1.22885E-05	0.001259574
Rps29	1.582396135	0.662110807	1.23129E-05	0.001259574
Slco1a4	-4.660992831	-2.220637294	1.23356E-05	0.001259574
Slc16a7	-1.609131019	-0.686281798	1.26278E-05	0.001280129
Synpr	-5.756066246	-2.525083196	1.27739E-05	0.001284288
Fam161b	-2.489086405	-1.315616312	1.28806E-05	0.001284288
Rgl1	-1.687963505	-0.755283712	1.29422E-05	0.001284288
Calcoco1	-1.600107548	-0.678168877	1.30917E-05	0.001290033
Upb1	-1.474767251	-0.560487285	1.32356E-05	0.001295162
Slco1a1	-2.420733476	-1.275444246	1.35952E-05	0.001321174
Ift122	-2.093220127	-1.065724036	1.4297E-05	0.001379852
Ccdc6	-1.860034927	-0.895329712	1.47988E-05	0.001418571
Mest	2.391786061	1.25808835	1.5103E-05	0.001437951
Ngef	-2.32938471	-1.219948928	1.52546E-05	0.001442636

Ppp1r14b	2.10878756	1.076413765	1.60706E-05	0.001509672
Pea15a	1.58300283	0.662663835	1.62771E-05	0.001518945
Gabarapl1	-1.817271101	-0.861773658	1.674E-05	0.001551866
Rpl36a	1.629085995	0.704062762	1.72626E-05	0.001589852
Lrp8	6.333751231	2.663060204	1.81895E-05	0.001664339
Arhgef39	-4.547947332	-2.185215547	1.86238E-05	0.001693083
Itga6	3.851573597	1.945447993	1.90502E-05	0.001720747
Tmem25	-2.446830298	-1.290914045	1.93565E-05	0.001737276
Cadps2	1.482705996	0.568232556	1.99008E-05	0.001774823
Ugt1a10	7.779841163	2.959740701	2.00303E-05	0.001775134
Tceal8	1.573924746	0.654366563	2.01653E-05	0.00177593
Dgkg	-2.259714984	-1.176140818	2.16046E-05	0.00189087
Tuba4a	2.080854867	1.057176345	2.23055E-05	0.001940163
Evpl	-2.602922768	-1.380132506	2.26154E-05	0.001955049
Ntrk3	2.052489521	1.037374856	2.29281E-05	0.001966265
Slc6a19	-1.690868279	-0.757764276	2.30242E-05	0.001966265
Cebpb	2.269059148	1.182094217	2.4007E-05	0.002037847
Mfsd7c	-3.821925298	-1.93429958	2.58717E-05	0.002182982
Klhl14	-2.523141646	-1.3352212	2.81877E-05	0.002364241
Acox2	-2.180728656	-1.12481027	2.89166E-05	0.00241103
Ciart	-3.264514465	-1.706868434	2.93275E-05	0.002430905
Agpat2	1.735530033	0.795376331	3.03168E-05	0.00249821
Cldn5	1.86950722	0.902658043	3.07735E-05	0.002521104
Pak3	4.240266015	2.084154776	3.16508E-05	0.00257782
Myh14	-1.573490394	-0.653968372	3.20898E-05	0.00257782
Tmem87b	1.99505768	0.996430458	3.2096E-05	0.00257782
Itih2	-3.547988112	-1.827001176	3.21976E-05	0.00257782
Acsm5	-2.196170772	-1.134990241	3.24438E-05	0.00258286
1700016C15	-1.622460485	-0.698183342	3.2673E-05	0.002586493
Gbe1	1.486110565	0.571541455	3.53723E-05	0.002784529
Mtmr7	-2.755931607	-1.462540086	3.71411E-05	0.002890209
Dlg2	-2.854975398	-1.513478314	3.72105E-05	0.002890209
Trpv2	5.010380604	2.324920199	3.73301E-05	0.002890209
Krt20	187.1850634	7.548321508	3.83095E-05	0.002938434
D630024D03	-2.221563635	-1.151575467	3.84027E-05	0.002938434
Xylt1	-2.615200522	-1.38692157	3.85785E-05	0.002938434
Cd63	1.533595838	0.616918327	4.29605E-05	0.003254608
Rps12	1.614018978	0.690657542	4.3232E-05	0.003257662
Idh1	1.669770862	0.739650139	4.34663E-05	0.003257893
Ppp1r1a	-1.82785515	-0.870151747	4.38825E-05	0.003271687
Jade3	1.79474155	0.843776105	4.53511E-05	0.003363383
Rbm3	2.449875427	1.292708392	4.56675E-05	0.003369115
Havcr1	9.790338828	3.29135879	4.60363E-05	0.003378631
Cul9	-1.952694195	-0.965466031	4.65283E-05	0.003388905
Tmem256	1.485757771	0.571198926	4.66573E-05	0.003388905
Treh	-1.516479291	-0.600725797	4.79152E-05	0.003462427
Ppt2	-1.810355681	-0.856273172	4.87804E-05	0.003496768
Trim13	1.628412297	0.703466021	4.88868E-05	0.003496768
Col6a6	-6.696018094	-2.743303426	5.03094E-05	0.003579676
Btbd11	-2.356235708	-1.236483867	5.05539E-05	0.003579676
Banp	-2.251677907	-1.171000471	5.19936E-05	0.00366321
Ctsd	1.580527835	0.660406443	5.2588E-05	0.003686654
Aaas	-1.690689187	-0.757611462	5.39196E-05	0.003761293
Flot1	1.738703612	0.798012025	5.49929E-05	0.003817265
Rpl12	1.633415881	0.707892159	5.53505E-05	0.003823252
Pde5a	-1.912737994	-0.935639268	5.58677E-05	0.003840155
Poli	-2.160657174	-1.111470182	5.64566E-05	0.003861796
2610015P09	-1.984887902	-0.989057532	5.75176E-05	0.003915362
Sec14l3	-3.965479087	-1.987495175	5.87298E-05	0.00396837
Lcn2	8.773925707	3.13322249	5.88595E-05	0.00396837
Cntnap5a	-3.85898527	-1.948221537	5.93786E-05	0.003974209
Maoa	1.620254606	0.696220535	5.95102E-05	0.003974209
Rsph3b	-1.457887811	-0.543879704	6.36896E-05	0.004227533
Emp1	2.117777751	1.082551194	6.43113E-05	0.004227533
Tmem108	2.620193732	1.389673486	6.4363E-05	0.004227533
Ttll5	-1.75240069	-0.809332688	6.45035E-05	0.004227533
Ucp2	2.163021943	1.113048301	6.55369E-05	0.004275376
Srxn1	5.148711333	2.364211386	6.69837E-05	0.004348622
Mkrn2os	1.555004719	0.636918958	6.7277E-05	0.004348622
Rras2	1.539499548	0.622461443	6.82368E-05	0.004390522
Gm1653	-4.072334523	-2.025856077	6.87696E-05	0.004404693
Npy6r	5.557921867	2.474545553	7.09029E-05	0.00451332
Tubb5	1.71656624	0.77952553	7.11062E-05	0.00451332
Ranbp3l	-3.162561493	-1.661093532	7.15584E-05	0.004521657
Ppp2r1b	1.707713048	0.772065575	7.25558E-05	0.00453526

Depdc1b	-2.094681039	-1.066730579	7.25661E-05	0.00453526
Naglu	-1.53172851	-0.615160611	7.27392E-05	0.00453526
Tuba1c	3.197911162	1.677129861	7.34366E-05	0.004558571
Mvd	-2.400746901	-1.263483315	7.40638E-05	0.004577336
Ptchd1	4.580765775	2.195588796	7.44544E-05	0.004581382
Tcea3	-1.77884617	-0.830941756	7.60125E-05	0.00465692
Rpl11	1.509260236	0.593841586	7.73587E-05	0.004712199
Cblc	-1.807800804	-0.854235721	7.75836E-05	0.004712199
Wdr19	-2.057597628	-1.040960884	7.82028E-05	0.004729421
Slc22a7	-12.83931664	-3.682496513	7.9102E-05	0.004763358
Zfp131	1.553349019	0.635382022	8.03944E-05	0.004816307
Bex4	1.922078608	0.94266734	8.09641E-05	0.004816307
Hsph1	-1.480460827	-0.566046317	8.10067E-05	0.004816307
Rpl22l1	1.691860776	0.758610854	8.38642E-05	0.004965253
Sec61g	1.518061681	0.602230411	8.45593E-05	0.004985463
Akr1c18	-18.3249222	-4.195735168	8.70442E-05	0.005110583
Rpl38	1.53582129	0.619010352	8.75243E-05	0.00511745
Dkc1	1.685511611	0.753186565	8.85838E-05	0.005157991
Cct6b	-2.645681209	-1.403639235	8.95716E-05	0.005194045
Tubb4b	1.916093766	0.938168162	9.08205E-05	0.005244885
Myo5a	-2.762245699	-1.465841652	9.13109E-05	0.005251679
Fads6	-2.535919963	-1.342509213	9.22863E-05	0.005286205
Apex1	1.591676261	0.670546928	9.28468E-05	0.005296776
Zfp36l1	4.376190216	2.129675448	9.71336E-05	0.005518993
Rgs2	2.232572793	1.158707215	0.000101348	0.005735316
Gclc	3.025009337	1.596939596	0.000104384	0.005883523
Gm853	-2.29840389	-1.20063234	0.000105249	0.005908634
Tspan13	-2.23149077	-1.158007839	0.000106089	0.00591644
Col23a1	-1.914137087	-0.936694157	0.000106228	0.00591644
Hsd3b4	-3.075890383	-1.62100409	0.000108581	0.00602368
Slc1a2	-2.405121632	-1.266109856	0.000110132	0.006081892
Ttc7b	-1.418714416	-0.504584207	0.000110494	0.006081892
Ptp4a1	2.639476785	1.400251977	0.000111882	0.006134355
Glud1	1.665025891	0.735544611	0.000113434	0.006195339
Tpk1	-1.573243205	-0.653741712	0.000115115	0.006258999
Tank	1.965542299	0.974927411	0.000115602	0.006258999
Bex1	2.792287568	1.481447527	0.000115932	0.006258999
Slc13a1	1.845498256	0.884010375	0.000116566	0.006269223
Txnrd1	2.484915347	1.313196705	0.000118051	0.006324907
Ccbl1	-1.943561286	-0.958702601	0.000118799	0.006329992
Rmdn2	1.80126913	0.849013752	0.000119044	0.006329992
Clec2h	-3.333940481	-1.737228349	0.000121495	0.006436023
Sox4	3.392291281	1.762260053	0.000122961	0.006468882
Rph3a	-11.846689	-3.566411996	0.000123033	0.006468882
Il5ra	-3.34593096	-1.742407677	0.0001243	0.00651117
Cltc	1.577288363	0.657446441	0.000125388	0.006543867
Lrrc59	1.863375339	0.897918305	0.000126447	0.006574794
Slc17a8	-3.644771493	-1.865828368	0.000127476	0.00660393
Fmod	-2.39888929	-1.262366578	0.000130191	0.006719861
Il13ra1	2.269152931	1.182153844	0.000130953	0.006734539
Slc2a12	-1.956344204	-0.968160224	0.000131478	0.006736913
Cdkl1	-2.121630899	-1.085173691	0.000132122	0.006745412
Hnrnpdl	1.916417307	0.938411748	0.000135061	0.006846702
Tsen54	-1.797374757	-0.845891245	0.000135078	0.006846702
Mir17hg	3.57584014	1.838282242	0.000136248	0.006881278
Aqp7	-2.586399535	-1.370945153	0.000137843	0.006936959
Fam217a	-2.193637431	-1.133325094	0.000139769	0.006993244
Grwd1	1.723311678	0.785183651	0.000140526	0.006993244
Nek4	-1.832346891	-0.873692653	0.000140611	0.006993244
Rap1b	1.809198474	0.855350684	0.000140947	0.006993244
Slc25a48	-2.098106934	-1.069088209	0.000142676	0.00704243
Cant1	-1.50464263	-0.589420871	0.000142938	0.00704243
Dis3	1.581564705	0.661352581	0.000144205	0.00708009
Wdr78	-2.005360795	-1.003861823	0.000147772	0.007230029
Tgfbi	2.009260367	1.006664526	0.000151081	0.007349546
Antxr1	1.698495743	0.764257602	0.000151257	0.007349546
Fga	5.909764181	2.563100563	0.000152822	0.007400064
Slc19a3	1.741688611	0.800486714	0.000160226	0.00773202
Fbxl21	-2.346726171	-1.23064951	0.000162975	0.007833964
Cdhr2	-1.71700933	-0.779897879	0.000163451	0.007833964
Rcan1	2.822326863	1.496885081	0.000164556	0.007860212
Rps25	1.496328091	0.581426541	0.000166482	0.007925341
Lnx2	2.060822063	1.043219944	0.000167694	0.007956167
Tgm7	-4.096239238	-2.034299978	0.000168314	0.007958777
Aplnr	-3.446078506	-1.784955568	0.000169755	0.008000077

Nim1k	-2.142213033	-1.099101957	0.000171357	0.00804864
Cd302	1.429610672	0.51562231	0.000173092	0.008103132
Ptger1	-3.679361026	-1.879455243	0.000177834	0.008297566
Tuba1b	1.679670369	0.748178135	0.00017945	0.008345294
Mfsd11	-1.593183837	-0.671912748	0.000180566	0.008369599
Rpl10a	1.510632482	0.595152713	0.000181681	0.00839364
Tsr1	1.765876901	0.820384777	0.000183238	0.008437943
Akr1b7	3.847670094	1.943985105	0.000185571	0.008466444
Tbc1d31	-2.456962487	-1.296875831	0.000185654	0.008466444
Cyfip2	-1.791523079	-0.841186629	0.00018566	0.008466444
2500002B13	-2.61973728	-1.389422139	0.000190757	0.008670842
Scap	-1.382031196	-0.466790181	0.000192089	0.008681019
Pwp2	1.592483562	0.67127848	0.000192618	0.008681019
Ehmt2	-1.404023919	-0.489567514	0.000192829	0.008681019
Ywhah	2.298202754	1.200506082	0.000195387	0.008756037
Selenbp2	-3.496451238	-1.805891385	0.000195739	0.008756037
Ppib	1.380524224	0.465216203	0.000199021	0.008874677
Sowahb	1.481283475	0.566847757	0.000200362	0.008880791
Pik3r2	-1.73830095	-0.797677876	0.000200418	0.008880791
Adora2b	10.21415618	3.352498119	0.000203445	0.008934119
Cdc42bpg	-1.364419349	-0.448287119	0.000203851	0.008934119
Frem2	-2.793655207	-1.482153974	0.000204041	0.008934119
Rpl35	1.444504169	0.530574367	0.000204288	0.008934119
Sv2a	-1.785662149	-0.836459146	0.000204792	0.008934119
Shank2	-2.473376379	-1.306481795	0.000212299	0.009221187
Usp2	-1.880060187	-0.910778848	0.000213373	0.009221187
Gas5	1.558225432	0.639903966	0.000214322	0.009221187
Cmtr2	1.9082571	0.932255559	0.000214615	0.009221187
Nek6	2.329885683	1.22025917	0.000215277	0.009221187
Afmid	-1.54750014	-0.629939539	0.000215779	0.009221187
Pde4c	-2.436797038	-1.284986093	0.000215953	0.009221187
Gnat1	5.246186737	2.391269161	0.000217075	0.009241109
D130020L05	-2.197273876	-1.135714704	0.000219702	0.009319444
Cacnb4	-2.215645162	-1.14772685	0.000220739	0.009319444
Mrps18b	1.789607539	0.839643239	0.000220899	0.009319444
Tmem184a	-1.522101033	-0.606064125	0.00022354	0.00940269
Rps15a	1.473187177	0.558940745	0.000230627	0.009671931
Sema6a	1.604195707	0.681850157	0.000231405	0.009675758
Srsf5	1.6295545	0.704477604	0.000232913	0.009709976
Asb9	1.594355606	0.672973445	0.000234351	0.009741123
Serpinb1a	2.584745148	1.37002204	0.000239573	0.009928908
Uap1	3.154319233	1.657328676	0.000241441	0.009976945
Abhd14b	-1.615531568	-0.692008942	0.000248005	0.010218224
17Rn6	-1.485771228	-0.571211994	0.000251665	0.010310121
Ndufc1	1.388478722	0.473505068	0.000251698	0.010310121
Rps21	1.476017834	0.561710153	0.000253212	0.01034205
Eif4b	-1.422954691	-0.508889725	0.000257216	0.010475242
Gspt2	1.880210788	0.910894409	0.000258312	0.010489553
Fads1	-1.578787649	-0.658817138	0.000259379	0.010494899
Cstb	1.815821958	0.860622753	0.000259933	0.010494899
Spp1	3.829882613	1.937300174	0.000263274	0.010531061
Oip5	-3.192906037	-1.674870097	0.000263474	0.010531061
Adarb1	-1.811028855	-0.856809533	0.000263664	0.010531061
Mttp	2.930135389	1.550967327	0.000263818	0.010531061
Cd9	1.842427269	0.881607669	0.000265798	0.010580096
Neurl1b	-2.184617319	-1.127380584	0.000267357	0.010609823
Acbd4	-1.757233632	-0.813306017	0.00026805	0.010609823
Ptbp2	1.486739454	0.572151843	0.000271276	0.010707428
Ndufa5	1.441298998	0.527369654	0.000272464	0.010724293
Pepd	-1.454933536	-0.54095325	0.000273362	0.01072965
Hmgb3	1.478393976	0.564030783	0.000274256	0.010734825
Scara3	2.408645875	1.268222301	0.000281316	0.010955168
Syn3	-3.070747332	-1.618589809	0.00028144	0.010955168
Cinp	1.424821487	0.510781178	0.000289786	0.011248965
Slc22a30	-1.723299779	-0.78517369	0.000291258	0.011275054
Mphosph6	1.710618603	0.774518134	0.000292962	0.011284389
Atf3	16.30530313	4.027269358	0.000293596	0.011284389
Sh3gl2	-1.684925621	-0.752684907	0.000293902	0.011284389
Upp2	-4.653354642	-2.218271142	0.000296065	0.01133657
Tspan4	1.729654065	0.790483525	0.000298796	0.01138354
Pxk	1.702416676	0.767584188	0.000299058	0.01138354
Slc25a24	2.317088295	1.212313021	0.000299716	0.01138354
Lrrc45	-1.970694841	-0.978704395	0.000306777	0.011620408
Acy3	-1.790517398	-0.840376537	0.000307691	0.011623781
Ugt1a2	8.883706258	3.151161691	0.00031007	0.011682356

Ccdc109b	2.317150727	1.212351892	0.00031144	0.011690755
Jmjd1c	2.368668632	1.244076385	0.000311953	0.011690755
1810011O10	1.667788741	0.737936554	0.000315903	0.011807404
Dgkz	-1.384139329	-0.468989173	0.00031751	0.01182076
Sav1	2.461487883	1.299530638	0.000317938	0.01182076
Proser3	-2.147299227	-1.102523245	0.000320569	0.011856343
1700113A16	-2.536936577	-1.343087453	0.000320578	0.011856343
Prrg4	11.14149673	3.47787115	0.00032381	0.011906998
Flot2	1.684594339	0.752401223	0.00032381	0.011906998
Ggnbp2os	-2.316826733	-1.212150154	0.000324483	0.011906998
Chchd1	1.460933405	0.546890416	0.000325962	0.011930201
Pcdh17	-1.514538832	-0.598878568	0.000328169	0.011967021
Ffar4	3.961789238	1.986152133	0.000328666	0.011967021
Pla2g6	-1.709899333	-0.773911392	0.000329558	0.011968571
Cask	1.457032287	0.543032847	0.000331557	0.011985167
Ift140	-2.198596196	-1.136582657	0.000331716	0.011985167
Clcf1	4.8424858	2.275747818	0.000338443	0.012196928
Sh3bgrl3	1.762714224	0.8177986	0.000340795	0.012250363
Pnpla7	-2.078596191	-1.055609513	0.000345258	0.012379198
Mt1	4.368482917	2.127132349	0.000349105	0.012458694
Dcdc2a	1.962091303	0.972392177	0.000349243	0.012458694
Trmt61a	1.796679778	0.8453333	0.000353829	0.012590424
Veph1	-2.862247324	-1.517148339	0.000355148	0.012593956
Rps20	1.527324084	0.611006221	0.000355916	0.012593956
Rab36	-2.358892639	-1.238109758	0.00035661	0.012593956
Sat1	2.193256324	1.133074428	0.000359123	0.012651018
Tbc1d1	1.464301693	0.550212826	0.000365487	0.01280782
Mkx	-4.881140775	-2.28721836	0.000365955	0.01280782
Golt1b	1.866463944	0.900307639	0.000366814	0.01280782
Rps27a	1.522366593	0.606315809	0.00036808	0.01280782
Rnd3	2.463819868	1.300896783	0.000368119	0.01280782
Klf12	-1.802054553	-0.849642686	0.000372272	0.012920405
Apol11b	4.591752827	2.199044984	0.000376126	0.013009117
Uqcrb	1.376180014	0.460669197	0.00037672	0.013009117
Ostc	1.384090475	0.468938252	0.000378646	0.013009117
Bbs2	-1.5425209	-0.625290037	0.000379923	0.013009117
4831440E17	-2.358485801	-1.237860915	0.000380194	0.013009117
Izumo4	-1.771726051	-0.825155548	0.000380367	0.013009117
Myc	3.594242871	1.845687898	0.000381648	0.013021322
Pcnt	-1.489604875	-0.5749297	0.000384125	0.013040308
Fam20c	-1.972776848	-0.980227774	0.000384329	0.013040308
Gorasp1	-1.643658237	-0.716910353	0.000386396	0.013040308
B930025P03	-11.41858914	-3.513312499	0.000386523	0.013040308
Uqcrh	1.394486109	0.479733563	0.000386832	0.013040308
Rpl23	1.434309994	0.520356864	0.000391758	0.013156852
Lama1	-1.563849382	-0.64510157	0.00039255	0.013156852
Mppe1	-1.719279323	-0.781803951	0.00039309	0.013156852
Skil	1.743768322	0.802208376	0.000398914	0.01332014
Mpped2	-1.942704192	-0.958066244	0.000400528	0.013342428
Ccdc114	-2.03783032	-1.027033931	0.000408848	0.013587458
Tmed5	2.504950229	1.324781939	0.000411809	0.013647242
Sntb1	2.34661326	1.230580094	0.000412584	0.013647242
Dnph1	-1.790868255	-0.840659209	0.000414797	0.013688298
Eif2s3y	1.514446111	0.598790243	0.00041965	0.013816101
Camk2d	1.622848834	0.698528621	0.000421417	0.013841942
Tnfrsf1a	1.976390722	0.982868188	0.000426716	0.013983399
Rnf24	-1.847682988	-0.885717251	0.000430841	0.014085809
Wfdc16	-4.995891081	-2.320742024	0.000432893	0.014120144
Tbxas1	-2.301439907	-1.202536773	0.000434067	0.01412572
Acp6	-1.819495653	-0.863538604	0.000435832	0.014150469
2310067B10	-1.789265596	-0.839367555	0.000441014	0.014268674
Klk1	1.458001985	0.543992684	0.000441498	0.014268674
Invs	-2.070445752	-1.049941403	0.000444934	0.014346824
Map3k4	-1.442115013	-0.528186229	0.000450835	0.014503932
Cryaa	-5.387491095	-2.42961358	0.000454673	0.014593775
Fus	1.729441645	0.790306335	0.000455699	0.014593775
Glis1	-2.011380415	-1.008185966	0.000456786	0.014595415
2510009E07	-1.430234621	-0.516251832	0.000460788	0.014658667
Cox7c	1.449455	0.535510544	0.000462128	0.014658667
Fads3	-1.525010605	-0.608819275	0.000463283	0.014658667
Snora31	2.912649993	1.542332346	0.000463853	0.014658667
Cmpk2	-4.192923064	-2.067956358	0.000463967	0.014658667
Tbc1d17	-1.35123255	-0.434275987	0.000468932	0.014755242
Impa2	-2.100681204	-1.070857237	0.000470075	0.014755242
Sdad1	1.520775125	0.60480684	0.000470166	0.014755242

Shfm1	1.436568809	0.522627097	0.00047213	0.014783963
Map3k2	2.032640634	1.023355173	0.000477335	0.014913813
Gbp6	-3.064921196	-1.61584998	0.000479724	0.014931766
Eif4a1	2.057189858	1.040674946	0.000480029	0.014931766
Ttc41	-2.335694871	-1.223851816	0.000481308	0.014938568
Zfp710	-1.416874187	-0.502711659	0.000489209	0.015150437
Ctps	1.839438153	0.87926517	0.000492801	0.015228202
Irx1	-1.549559121	-0.631857799	0.000494179	0.015237359
Baiap2	-1.789643379	-0.839672131	0.000497393	0.01530297
Sumo2	1.357812676	0.441284459	0.000499815	0.015314082
Lgmn	1.585142285	0.664612345	0.000499927	0.015314082
Socs3	4.755770153	2.249678991	0.000502701	0.015365638
Dgkb	-1.896593521	-0.923410512	0.000508031	0.015494931
Kif27	-3.833422963	-1.938633188	0.000509785	0.015514871
Grrp1	1.742132048	0.80085398	0.000519902	0.015777173
Mustn1	1.783242159	0.83450263	0.000520643	0.015777173
Plp2	1.606339504	0.683776842	0.000522617	0.015777279
Sema4g	-1.784768211	-0.835736723	0.000522886	0.015777279
Mapkapk2	2.149000042	1.103665511	0.000526243	0.015844397
Gm20554	-2.133481202	-1.093209399	0.000527796	0.015844397
Slc20a2	1.516420471	0.600669837	0.000528484	0.015844397
Susd2	-1.938855409	-0.955205218	0.000535625	0.016024395
Lrp4	-1.781284233	-0.83291774	0.000537453	0.01604502
Mydgf	1.410655271	0.496365472	0.000539806	0.016074679
Arid5a	5.25757591	2.394397775	0.000540728	0.016074679
Tcerg1	1.64091843	0.714503525	0.000545242	0.016125497
Acat3	1.882132698	0.912368347	0.0005458	0.016125497
Hint1	1.440837973	0.526908109	0.00054589	0.016125497
Dyx1c1	-2.3704013	-1.245131322	0.000547015	0.016125497
4930556M19	-2.379862298	-1.2508781	0.000552502	0.016218187
Klk1b16	3.789659865	1.922068368	0.000553502	0.016218187
Txn1	1.578159124	0.658242678	0.000554926	0.016218187
Rpl9	1.415196118	0.501001996	0.000555981	0.016218187
Plxdc2	-1.511464465	-0.595947061	0.000556189	0.016218187
Afap1l1	-1.596961375	-0.675329419	0.000557065	0.016218187
Rrp1b	1.751474466	0.808569956	0.000561746	0.016320748
Pxylp1	-1.7977779	-0.846214799	0.000566329	0.016420037
Apln	-2.584338556	-1.369795079	0.000567961	0.016433544
Rpl36al	1.380024067	0.464693427	0.000569156	0.016434377
Slc33a1	-1.614377449	-0.690977927	0.000573216	0.016517757
Wdr34	-1.467854738	-0.553709203	0.000575909	0.016561489
Gal3st1	-1.672915115	-0.742364244	0.000580016	0.016591188
Apeh	-1.705976095	-0.770597431	0.00058014	0.016591188
Acmsd	-1.820321783	-0.864193502	0.000580474	0.016591188
Cyp3a13	4.086020992	2.030696616	0.000584125	0.016661747
Ppfibp1	1.867243547	0.900910113	0.000587843	0.016711504
Rec8	-2.689381392	-1.427274364	0.000588241	0.016711504
Acsm1	-2.087854456	-1.062021145	0.000592586	0.016801053
Slc22a19	-2.5098244	-1.32758643	0.000596919	0.01684175
Rpl24	1.426899707	0.512883936	0.000597055	0.01684175
Fam122b	-1.712712748	-0.776283207	0.000597607	0.01684175
Rps16	1.567306405	0.648287251	0.00060209	0.016934224
Mc2r	2.649992682	1.405988376	0.000604203	0.016959799
Fam107a	-2.308875253	-1.207190227	0.000611638	0.017134373
Dym	-1.517481501	-0.601678929	0.000615231	0.017177452
Nras	1.902064418	0.927566107	0.000615614	0.017177452
Lrrc46	-3.731490778	-1.899752121	0.000617638	0.017199863
Mep1a	-1.419514953	-0.505398046	0.00062037	0.017241893
Rhbdd3	-1.657998161	-0.729442407	0.000622502	0.017267088
Btg2	5.522047054	2.465203181	0.000634131	0.017555085
Amacr	-2.131573205	-1.091918603	0.000636917	0.017597646
Junb	3.044666343	1.606284136	0.00064002	0.017648763
Kcne4	5.471533564	2.451945249	0.000644598	0.017705721
Zfp296	-4.153005887	-2.054155917	0.000644598	0.017705721
Srcin1	-2.928502829	-1.550163288	0.00064724	0.017722814
Rpl36	1.528084999	0.611724795	0.000647736	0.017722814
Gm3716	-6.690886105	-2.742197286	0.000650351	0.017759874
Exoc3l	-2.158957534	-1.110334866	0.000653313	0.017806263
Cep164	-1.601813552	-0.67970623	0.00065644	0.017856931
Alcam	1.3847155	0.469589594	0.000658243	0.01787148
Slc25a35	-2.088947615	-1.062776314	0.000676485	0.018331453
Rpl35a	1.439503644	0.525571441	0.000678256	0.018344169
Tyk2	-1.570021074	-0.650783924	0.000679571	0.018344506
Ascc1	-1.574395393	-0.654797904	0.000688301	0.018491678
Sdc1	3.407620801	1.768764802	0.000688861	0.018491678

Ndufa2	1.34824601	0.431083765	0.00068896	0.018491678
Zbed6	1.584531762	0.664056579	0.000691135	0.018514787
Gpatch4	1.500744506	0.585678387	0.000694684	0.018574557
Ypel2	3.160309958	1.660066062	0.000699565	0.01866965
Ptpn3	-1.352807717	-0.435956795	0.000715965	0.019071197
Glce	1.681368835	0.749636238	0.000720442	0.019140012
Mgmt	-1.735851733	-0.795643726	0.000721265	0.019140012
Snhg3	1.797011916	0.845599975	0.000724307	0.019179067
Rps5	1.469607452	0.555430847	0.00072553	0.019179067
Agt	1.739959452	0.799053686	0.00072682	0.019179067
Rpl31	1.390622081	0.475730403	0.000729974	0.01922057
Smim5	-3.914286496	-1.968749354	0.000731694	0.01922057
Nop58	1.910852152	0.934216158	0.000732485	0.01922057
Edar	-5.096418921	-2.349483871	0.00073921	0.019318391
Igf2bp2	1.692929578	0.759521961	0.000740177	0.019318391
Fam78a	-3.425944864	-1.776501933	0.000740416	0.019318391
Klkb1	-2.229179402	-1.156512728	0.000741697	0.019318391
Syde1	1.516624176	0.600863625	0.000743644	0.019333382
Lipo1	-1.761448895	-0.816762618	0.000747684	0.019402596
Tgif1	4.250838974	2.087747609	0.000758224	0.019635309
Acaa2	2.227878223	1.155670376	0.000759438	0.019635309
Rrp15	1.553382429	0.635413052	0.000776009	0.020026999
Insl6	1.716630831	0.779579815	0.000780073	0.020043884
Map4k2	-1.431995351	-0.518026808	0.000780312	0.020043884
Atp13a2	-1.60147931	-0.67940516	0.000781624	0.020043884
Adamts4	5.534431831	2.468435216	0.000782353	0.020043884
Rassf4	-2.453863362	-1.295054918	0.000785986	0.020084857
Ankrd13c	-1.560619885	-0.642119187	0.000786803	0.020084857
Engase	-1.986977383	-0.990575451	0.000788436	0.020090136
Slirp	1.348756845	0.431630281	0.000797564	0.020286047
Klf6	5.277439787	2.399838213	0.000799952	0.02031014
Lpin1	-1.876987459	-0.908419011	0.000803744	0.020368989
Calml3	3.321817659	1.731972883	0.000805161	0.020368989
Cenpe	-2.396229726	-1.260766226	0.000807981	0.020403684
Elmsan1	2.025754202	1.018459133	0.000812655	0.0204491
Ier3	2.897523474	1.534820349	0.000812682	0.0204491
Emd	1.836575533	0.877018231	0.000816662	0.020512621
Tsc22d1	2.472084117	1.305727834	0.000819082	0.020536806
Fam151a	-1.938972911	-0.955292648	0.000824295	0.020614248
Fbxo36	-1.479199104	-0.564816256	0.000825624	0.020614248
Kcnc3	-1.699984063	-0.765521221	0.000827654	0.020614248
Ppp1r1b	-3.057371028	-1.612291642	0.000828399	0.020614248
Rap2b	9.312815708	3.219217429	0.000829485	0.020614248
Katnal1	-1.773675499	-0.826742087	0.000834827	0.020710464
Sympk	-1.42927372	-0.515282234	0.000840028	0.020785652
Gusb	-1.454546879	-0.540569794	0.000841999	0.020785652
Sfn	3.334862348	1.737627213	0.00084256	0.020785652
Cpb2	-3.842209697	-1.941936259	0.000843758	0.020785652
Evc2	-1.67412564	-0.743407803	0.000847033	0.02080263
Rpl5	1.458701836	0.544685021	0.0008474	0.02080263
Coprs	-1.819805243	-0.86378406	0.000858872	0.021038486
Pdpn	1.652658721	0.724788834	0.000859993	0.021038486
S100a11	1.675500329	0.74459197	0.000876215	0.021392447
Pdk4	2.465245257	1.301731181	0.000877499	0.021392447
Rgl3	-1.583486607	-0.663104665	0.000882136	0.021445137
Larp4	1.485727598	0.571169628	0.000883	0.021445137
B4galt6	1.924164451	0.944232106	0.000884226	0.021445137
Panct2	-2.163532743	-1.113388954	0.000887082	0.021477452
Rps8	1.434751494	0.520800877	0.000889211	0.021492073
Ankrd50	1.639085317	0.712890951	0.00089258	0.021536555
Fmo5	-2.267810727	-1.181300237	0.000894377	0.021543014
Ddx3x	1.485363727	0.570816253	0.000901479	0.02165305
Chmp2b	1.47270018	0.558463749	0.000902018	0.02165305
Pvrl4	2.044685354	1.031878852	0.000930905	0.022308472
R3hdm2	-1.306750962	-0.385984222	0.000934681	0.02235579
Cirbp	1.615799231	0.692247949	0.000936053	0.02235579
Magoh	1.410295843	0.495997834	0.000937834	0.022360427
Prdx4	1.588097119	0.667299142	0.000942593	0.022390468
Cep89	-1.451315478	-0.537361157	0.000942719	0.022390468
1600014C10	-2.320212424	-1.214256895	0.000944922	0.022390468
Fbxo40	-3.180559686	-1.66928066	0.000945449	0.022390468
Ankrd42	-1.698768467	-0.764489234	0.000948714	0.022430086
Gm166	-1.911979198	-0.935066827	0.000952003	0.022470132
Hilpda	3.741097905	1.903461722	0.000954454	0.022490329
Grtp1	-2.425477782	-1.278268964	0.000960605	0.022597481

Srsf3	1.657892832	0.729350753	0.000968835	0.022753079
Saa2	24.06464179	4.588843043	0.000971689	0.022782154
Ngfrap1	1.483322804	0.568832594	0.000973593	0.022788867
Elavl3	-2.701260568	-1.433632811	0.00098193	0.022945888
Trnp1	-2.978898688	-1.574779058	0.000995968	0.023197003
Sec22c	-1.749609	-0.807032546	0.000995968	0.023197003
Ank3	-1.42188274	-0.507802494	0.001013878	0.023542815
Bhlhe40	1.855535172	0.891835348	0.001015181	0.023542815
Phlda1	3.79156706	1.92279424	0.001015828	0.023542815
Ankmy2	-1.740548269	-0.799541824	0.001021861	0.023643759
Lysmd3	1.931256013	0.949539425	0.001028418	0.023756444
Cd14	4.763144769	2.251914398	0.001032937	0.023821784
Rps19	1.403016287	0.488531757	0.001036155	0.023856962
Tnfsf10	-1.904316839	-0.929273533	0.001038091	0.023862549
Rpl32	1.432399074	0.518433491	0.001043113	0.023938936
Rnf167	-1.295873068	-0.373924412	0.001053632	0.024094741
Tsga10	-1.863817644	-0.898260713	0.001054088	0.024094741
Dlec1	-1.507781542	-0.592427416	0.001057044	0.024094741
Prkag2	1.499430449	0.584414605	0.001057732	0.024094741
Fam171a2	-2.160406404	-1.11130273	0.001058671	0.024094741
Hk2	4.700973255	2.232959473	0.001061583	0.024094741
Ost4	1.377058036	0.461589363	0.001064066	0.024094741
Rps24	1.40266978	0.488175406	0.001065593	0.024094741
Smc2	-1.647070481	-0.719902292	0.001066874	0.024094741
Kl	-1.65995694	-0.731145818	0.001068596	0.024094741
Efhd1	-1.58924111	-0.668338018	0.001070471	0.024094741
Pde4b	2.472056946	1.305711977	0.00107172	0.024094741
Ccnl1	2.018833239	1.013521745	0.001072131	0.024094741
Fam53b	-1.845864812	-0.884296896	0.001085856	0.024364334
Lamc2	2.015832207	1.011375557	0.001087898	0.02437133
Slc39a1	1.547280216	0.629734496	0.001096673	0.024528913
Nmd3	1.384105099	0.468953495	0.001105346	0.024683724
Nid2	-1.483361927	-0.568870645	0.001107892	0.024701444
Pax2	-1.71622132	-0.779235611	0.001112282	0.024760138
Etnppl	-1.629746523	-0.704647598	0.001114128	0.024762112
Ypel5	1.549826427	0.63210665	0.001119159	0.024815229
Pcdhb17	2.013357545	1.009603398	0.00112004	0.024815229
Cox6b1	1.384981024	0.46986621	0.001138498	0.025151085
Bzw1	1.523727744	0.607605149	0.001140524	0.025151085
Pde6d	-1.657264352	-0.728803747	0.001140554	0.025151085
Gnmt	-1.596789109	-0.675173785	0.001158054	0.025457136
Lrrc52	-2.493485044	-1.31816355	0.001158999	0.025457136
Nifk	1.384985719	0.4698711	0.001159852	0.025457136
Bmf	1.848287715	0.886189353	0.001164814	0.025526276
Hook2	-1.650404518	-0.722819676	0.001173629	0.025679502
BC089597	2.801516045	1.486207755	0.001178436	0.025744706
Errfi1	1.691162612	0.758015387	0.001181266	0.025766597
Galnt7	-1.421680141	-0.507596915	0.001185933	0.025828416
Tmem174	-1.961519816	-0.97197191	0.00119465	0.025941737
Tpt1	1.516779511	0.601011381	0.00119577	0.025941737
Cxcl9	-7.066257601	-2.820946344	0.00119666	0.025941737
Aifm3	-1.770579687	-0.824221776	0.001204072	0.02606234
Ppp1cc	1.481172812	0.566739973	0.001206283	0.026070149
Sec22b	1.601630015	0.679540916	0.001208789	0.02608429
Akip1	-1.639253237	-0.713038744	0.001215956	0.026156885
Abhd11os	-1.781454227	-0.833055415	0.001216951	0.026156885
Tnfrsf12a	4.437572021	2.149770534	0.001217722	0.026156885
Lman2l	-1.725428693	-0.786954853	0.001224579	0.026264135
Gne	-2.130052976	-1.090889312	0.001235179	0.026451236
Gpr179	-3.388191455	-1.760515399	0.001243657	0.02657189
Cys1	-2.37730739	-1.249328458	0.001245202	0.02657189
Zwint	1.672534253	0.742035757	0.001246471	0.02657189
Ppia	1.382081259	0.466842441	0.001249729	0.026601105
Col6a4	-3.885678534	-1.95816655	0.001254393	0.026652371
Kras	2.070588596	1.050040934	0.001257862	0.026652371
Rnpep	-1.480032357	-0.565628717	0.001258227	0.026652371
Naa15	1.410182297	0.495881675	0.001260741	0.026652371
Ddx39	1.616852873	0.693188405	0.0012627	0.026652371
Brix1	1.399821093	0.485242453	0.00126509	0.026652371
Tatdn2	1.396923947	0.482253478	0.001266077	0.026652371
Srm	1.478248215	0.563888535	0.001267269	0.026652371
Acad12	-1.531320077	-0.614775867	0.00127068	0.026682572
Mapk4	1.914116301	0.93667849	0.001272492	0.026682572
Prdx1	1.391064175	0.476188979	0.001274449	0.026683892
Ptpmt1	1.434641664	0.520690434	0.0012789	0.026734482

Arhgef16	-1.300626813	-0.379207071	0.00128066	0.026734482
Gpm6b	1.920768076	0.941683331	0.001284687	0.026774629
Tax1bp3	1.687164763	0.754600869	0.001289081	0.026774629
Rcan3	-1.487965236	-0.573340821	0.001289624	0.026774629
Tctn3	-1.483879007	-0.569373462	0.001290183	0.026774629
Cav2	1.315328128	0.395422746	0.001295374	0.02684281
Myo5b	1.345933041	0.428606639	0.00130716	0.027019611
Scn5a	2.874432663	1.523277234	0.001307741	0.027019611
Tmc5	2.021327716	1.015303244	0.001317304	0.027177345
4933412E12	-2.0024893	-1.001794534	0.001327159	0.027340642
1110019D14	-2.259266076	-1.175854188	0.001334175	0.027445054
Mpdz	-1.427501887	-0.513492652	0.001339529	0.027465988
Serpina3k	-15.67365797	-3.970270015	0.001339703	0.027465988
9030624J02	-1.441038731	-0.527109112	0.00134104	0.027465988
Arsb	-1.423647747	-0.509592225	0.001352176	0.027611915
Chrnb2	-1.994674217	-0.996153136	0.001353037	0.027611915
Abcb1a	1.848188434	0.886111855	0.001354044	0.027611915
Aspa	-1.65308629	-0.725162035	0.001360781	0.027700061
Parp3	-1.409989098	-0.495684008	0.001364356	0.027700061
Prlr	4.520337256	2.176430414	0.001365202	0.027700061
1810014B01	-2.216702181	-1.148414954	0.00136623	0.027700061
Ttc38	-1.632062978	-0.706696729	0.001373423	0.027775064
Efs	-3.442311473	-1.783377644	0.001373871	0.027775064
Dad1	1.353411197	0.43660023	0.001390924	0.028064826
Gpr85	-3.105779289	-1.634955309	0.001393464	0.028064826
Oas1c	-2.089124281	-1.06289832	0.001394179	0.028064826
Asap3	-1.683278107	-0.751273555	0.00140045	0.028150843
Id1	1.810273916	0.856208011	0.001405349	0.028174668
Pprc1	2.008826848	1.006353215	0.001405634	0.028174668
Efcc1	-4.932305683	-2.302262215	0.001414224	0.028306576
Mboat1	2.330698174	1.220762187	0.001422453	0.028430902
Ube2u	-1.85878769	-0.894361996	0.001425642	0.028454286
Slc22a3	3.229145416	1.69115241	0.001427715	0.028455354
Gm16157	-3.079053876	-1.622487111	0.001437989	0.028600082
Tbx10	-3.282105178	-1.714621472	0.001439036	0.028600082
Bmper	2.652999692	1.407624508	0.001466037	0.029095667
Jund	2.479008036	1.309762948	0.001471932	0.029171583
Phyhd1	-1.375657078	-0.460120882	0.001474728	0.02918595
Evc	-1.929791859	-0.948445252	0.001480592	0.029260896
Rbl1	-1.712189038	-0.775841995	0.001491604	0.029400201
Rlim	1.598841947	0.677027329	0.001491813	0.029400201
Bspry	-1.424120996	-0.510071726	0.001499688	0.02951411
Calm2	1.506652506	0.591346713	0.001501802	0.029514498
Pi4ka	-1.560541332	-0.642046569	0.00150587	0.029542288
Sema6b	1.876975125	0.908409531	0.001508193	0.029542288
Rpl3	1.372154178	0.456442594	0.001510724	0.029542288
4933406C10	-2.652313513	-1.407251317	0.001513674	0.029542288
Tfdp2	-1.424796568	-0.510755946	0.001513699	0.029542288
Cldn8	-1.588577231	-0.66773523	0.001522962	0.029681964
Spice1	-1.778778163	-0.830886599	0.001525336	0.029687162
Slamf7	-2.369362242	-1.244498783	0.001535755	0.029848723
H3f3b	2.033035307	1.02363527	0.001543076	0.029949699
C3	3.258130317	1.704044309	0.001546658	0.029977941
Endod1	1.436475689	0.522533577	0.00154961	0.029993894
Stradb	-1.527119662	-0.610813113	0.001552855	0.030008545
Zfp85os	-2.91004129	-1.541039624	0.001555424	0.030008545
Il20rb	2.963231189	1.567171189	0.001558522	0.030008545
Rpain	1.3737877	0.458159073	0.001560902	0.030008545
Neurl4	-1.430503469	-0.516522997	0.001563108	0.030008545
Ezr	2.646463444	1.404065726	0.001563145	0.030008545
Arl15	1.680381097	0.748788462	0.001572463	0.030114966
Gstt3	-1.438515418	-0.524580684	0.001572963	0.030114966
D630029K05	-2.739289471	-1.453801729	0.001578903	0.030121177
Heatr1	1.636896048	0.710962705	0.001580866	0.030121177
Rps10	1.385600088	0.470510927	0.001581294	0.030121177
Phf13	2.156038988	1.108383267	0.001581837	0.030121177
Hadhb	1.419396211	0.50527736	0.001598641	0.030400073
Kcnma1	-1.697821077	-0.76368443	0.001600926	0.030402494
Pcp4l1	1.929896517	0.948523491	0.001606561	0.030468449
Map3k14	2.631558077	1.395917234	0.001613792	0.030564439
Bcl2l1	1.740872266	0.799810351	0.001617801	0.03059924
Ush1c	-1.495817797	-0.580934453	0.001624128	0.030677723
Nrip2	-2.361436045	-1.239664463	0.001635833	0.03075724
Paqr7	-1.814093103	-0.8592485	0.001636317	0.03075724
Polr2h	1.606973631	0.684346256	0.001639979	0.03075724

Rpl18	1.395433123	0.480712984	0.001640689	0.03075724
Pmepa1	1.574896948	0.65525743	0.001642695	0.03075724
F3	3.888572105	1.959240491	0.00164279	0.03075724
Tpgs2	-1.473057258	-0.558813509	0.001643617	0.03075724
Ifit3b	-4.733385069	-2.242872293	0.001659101	0.030988991
Rap1gap	-1.990326969	-0.993005455	0.001660399	0.030988991
Slc51b	1.864389736	0.898703476	0.001664847	0.030998395
Asah2	-1.384305761	-0.469162636	0.001665303	0.030998395
Osbpl6	-1.329972302	-0.411396201	0.001682269	0.031253226
Abce1	1.366727352	0.450725469	0.001683429	0.031253226
Cox20	1.544288285	0.626942098	0.001688071	0.031298177
Sytl1	-1.988620915	-0.991768286	0.00169219	0.031333303
Ppan	1.663347162	0.734089309	0.001694919	0.031342653
Nupr1l	-2.687147627	-1.426075582	0.00170218	0.031407318
Sectm1a	-3.391864704	-1.762078624	0.001702874	0.031407318
Zfp593	1.980796505	0.986080674	0.001707743	0.031455959
Col13a1	2.673334571	1.418640404	0.001713901	0.031501675
Defb1	2.286946339	1.193422515	0.001714696	0.031501675
Phldb1	-1.392988365	-0.478183207	0.001719774	0.031553817
Timm23	1.358195228	0.441690868	0.001733837	0.031770478
Gm3219	1.881601188	0.911960876	0.001736709	0.031781775
Elovl5	1.308422279	0.38782823	0.001760499	0.032175344
Rnls	-2.53216313	-1.340370351	0.00176338	0.032186259
Abracl	1.567459802	0.648428446	0.001767571	0.032215886
Dcst1	-2.239758706	-1.163343316	0.001769791	0.032215886
Mif	1.41528975	0.501097443	0.001771862	0.032215886
Tmem258	1.402604093	0.488107843	0.001784674	0.032399901
Ngf	2.346106421	1.230268457	0.001786582	0.032399901
Ier2	3.001146434	1.585513713	0.001795256	0.032515369
Pcdh7	-3.949804886	-1.981781388	0.001797883	0.032521145
Sugp1	-1.341748822	-0.424114621	0.001804686	0.032602339
Cndp1	-1.863443127	-0.897970788	0.001807884	0.032603195
Tjp2	2.008577365	1.006174031	0.001811189	0.032603195
Dcaf4	-1.999615826	-0.99972285	0.001811674	0.032603195
Rpl37	1.439076764	0.525143551	0.001814077	0.032604789
Rpl23a	1.37800054	0.462576454	0.001828071	0.032789521
Coq7	1.360606855	0.444250263	0.001829009	0.032789521
Dhx34	-1.623337029	-0.698962556	0.001833916	0.032812322
Otulin	1.381566905	0.466305429	0.001834938	0.032812322
Gm11127	-1.837905266	-0.878062405	0.001844687	0.032910719
Usp8	-1.3454797	-0.428120624	0.001845112	0.032910719
Litaf	2.813182878	1.492203342	0.001851849	0.03298914
Actg1	1.93831813	0.954805375	0.001856539	0.033030926
Rps4x	1.375370123	0.459819912	0.001879207	0.033392062
Acot10	3.277990179	1.712811532	0.001885455	0.033418229
Eef1e1	1.583404788	0.663030119	0.001885848	0.033418229
Snrpg	1.481574384	0.56713106	0.001892228	0.033418229
Klhl32	-2.312430974	-1.209410302	0.001893509	0.033418229
Pcsk9	2.592598108	1.374398584	0.001893923	0.033418229
Thra	-1.434836131	-0.52088598	0.001897647	0.033418229
Hpd	-2.486011969	-1.313833242	0.001902085	0.033418229
Mcoln2	3.175992375	1.667207449	0.001902418	0.033418229
Stk17b	1.693752106	0.760222741	0.00190272	0.033418229
Slc6a12	-1.907027803	-0.931325878	0.001904708	0.033418229
Ces2h	-2.253499512	-1.172167138	0.001906767	0.033418229
Gnai3	1.506528656	0.591228115	0.001910048	0.03343414
Gan	1.884195037	0.913948309	0.001915491	0.033487827
Yif1b	1.337342243	0.419368717	0.001926343	0.033635811
Cpne8	1.968559015	0.977139964	0.001935916	0.033761128
Pak4	-1.308492103	-0.387905218	0.001940551	0.033800135
Gbp4	-3.20448335	-1.680091774	0.00196086	0.034107893
Mbd4	-1.912990081	-0.935829394	0.001966443	0.034107893
Cox7a2	1.34649838	0.429212494	0.001968506	0.034107893
Gale	1.843773905	0.882661755	0.001971305	0.034107893
Sorbs1	1.685601951	0.753263889	0.001973223	0.034107893
Usp24	1.341459648	0.423803658	0.001974421	0.034107893
Ift52	-1.445295579	-0.53136457	0.001975164	0.034107893
Tmcc3	2.434227932	1.283464263	0.001978263	0.034119584
Eef1b2	1.360994089	0.4446608	0.001985157	0.034196641
Rpl19	1.390008748	0.475093963	0.001997673	0.034370223
Cyp4f17	3.057748558	1.612469777	0.002002817	0.034416694
Abcc6	-1.604354465	-0.681992925	0.002012485	0.034474838
Rps6ka3	1.626614859	0.701872698	0.002013139	0.034474838
Atp5sl	-1.380259764	-0.464939807	0.00201354	0.034474838
Tmem56	-1.363362893	-0.447169623	0.002019831	0.034512267

Prosc	-1.526028129	-0.609781555	0.002020625	0.034512267
Ncapd2	-1.715020121	-0.778225503	0.00202839	0.034597812
Egr1	6.182759319	2.628250846	0.002032998	0.034597812
Phtf2	-1.408826557	-0.49449401	0.002033931	0.034597812
S100a10	2.386122857	1.254668327	0.002035768	0.034597812
Ankrd2	3.582863868	1.841113229	0.00203791	0.034597812
Odc1	-1.844908497	-0.883549264	0.002048767	0.034740287
Mmadhc	1.342848299	0.425296334	0.002069091	0.035042735
Zer1	-1.588031726	-0.667239735	0.002078082	0.035152766
Tnfrsf26	-2.107357628	-1.075435167	0.002081477	0.035167972
Nlrx1	-1.613288599	-0.690004543	0.002087535	0.03522809
2810408A11	-2.414843969	-1.271929975	0.002095257	0.035305001
Tpm3	1.840562092	0.88014642	0.002097409	0.035305001
Ssr1	1.292323129	0.369966843	0.002099609	0.035305001
Gch1	5.140297787	2.36185194	0.002103247	0.035321195
Tiparp	3.564767551	1.833808005	0.002105585	0.035321195
Snx21	-1.540988857	-0.62385643	0.002111928	0.035345191
Abcd1	-1.657488484	-0.728998846	0.002112033	0.035345191
Gys2	-2.151185878	-1.105132189	0.002117517	0.035394937
Snrpd1	1.567409602	0.64838224	0.00212146	0.035418831
Tfap2b	-1.688646652	-0.755867477	0.002127232	0.035473164
Ell2	2.043912325	1.031333312	0.002166901	0.036074591
Hif1a	1.6547109	0.726579181	0.002168418	0.036074591
Sepw1	1.281168128	0.357459814	0.002173896	0.03607651
Rpl27a	1.576420824	0.656652712	0.002174116	0.03607651
Hp1bp3	-1.268038516	-0.342598567	0.002176229	0.03607651
Aspn	-2.363839317	-1.241131971	0.002178774	0.03607651
Gng5	1.503232519	0.588068181	0.002188934	0.036200038
Slc4a1	-4.678142199	-2.225935715	0.002191373	0.036200038
Id3	2.288869077	1.194634943	0.002203095	0.036351061
Lzts3	-2.367454862	-1.243336919	0.002208014	0.036389616
Rbl2	-1.536370586	-0.619526249	0.002215481	0.036454811
Nqo1	1.835634596	0.876278902	0.002217144	0.036454811
1810022K09	1.446011685	0.53207921	0.002226371	0.03655444
Insig1	-1.769110213	-0.823023929	0.002230165	0.03655444
Iqce	-1.722457108	-0.784468058	0.002232001	0.03655444
Yrdc	1.852386897	0.889385457	0.00223358	0.03655444
Bsnd	-2.445581183	-1.290177357	0.00224401	0.036682533
Efemp1	1.676085434	0.745095689	0.002247464	0.036696428
Pgd	1.948048129	0.962029321	0.002252861	0.036741969
Dhcr7	-1.779916156	-0.831809284	0.002258345	0.036750285
Fam45a	1.27175162	0.346816931	0.002258587	0.036750285
Xrcc1	-1.54668028	-0.629175003	0.002264245	0.03679985
Mapk1ip1	-1.405412691	-0.490993832	0.002271385	0.036873369
Lekr1	-1.753112695	-0.80991874	0.002276275	0.036910229
L1cam	-1.462377522	-0.5483158	0.002281581	0.036953752
Lsm8	1.537045675	0.620160037	0.0022869	0.036997372
Btbd2	-1.675301931	-0.744421129	0.002297357	0.037123922
Abhd14a	-1.588482826	-0.667649492	0.002304958	0.037204084
Prkrir	1.39069056	0.475801444	0.002326478	0.037491341
Cd151	1.62492811	0.700375892	0.002328076	0.037491341
Bmp7	-1.381115369	-0.465833838	0.002343877	0.037702701
Rps17	1.384214994	0.469068038	0.002354322	0.037767178
Eddm3b	-10.44847143	-3.385219992	0.002356708	0.037767178
Gt(ROSA)26	1.645112348	0.718186112	0.002357907	0.037767178
Wasf3	-1.765412046	-0.820004947	0.002358606	0.037767178
Fam69b	-2.633839141	-1.397167237	0.002363246	0.037798526
Mir22hg	1.47741319	0.563073363	0.002366982	0.037815359
Vps37b	1.817172905	0.861695699	0.002370263	0.037824882
Fbxw15	-3.401753291	-1.766278515	0.002373361	0.037831481
Nckap5	1.606532837	0.683950469	0.002391144	0.037999472
Kif9	-1.589033201	-0.668149268	0.002395316	0.037999472
Soat1	-1.384520271	-0.469386177	0.00239581	0.037999472
Zfp948	1.819029034	0.863168571	0.002396254	0.037999472
Nfe2l2	1.562930363	0.6442535	0.002397384	0.037999472
Hnrnpab	1.491818867	0.577072377	0.002403199	0.038048852
Acot8	1.384005282	0.468849449	0.002430067	0.038431059
Vkorc1l1	1.376817973	0.461337835	0.002438926	0.038492873
Kcnk1	1.526613786	0.610335125	0.002439439	0.038492873
1110007C09	1.774196398	0.82716572	0.002458096	0.038712625
Prkab1	1.573245495	0.653743812	0.002458996	0.038712625
Cdr2l	2.565966538	1.359502357	0.002461608	0.038712625
Pdgfb	1.422332031	0.508258289	0.002483648	0.038975801
Arl4c	1.880802782	0.911348578	0.002483874	0.038975801
Cyp51	-2.294354294	-1.19808819	0.002499651	0.039162897

Dclk3	-2.085644832	-1.0604935	0.002502079	0.039162897
Wscd2	-3.854811705	-1.946660391	0.002504135	0.039162897
Fbxo21	-1.774075232	-0.82706719	0.002515446	0.039270834
6820408C15I	-2.38959745	-1.256767603	0.002516611	0.039270834
Idi1	-2.397489651	-1.261524588	0.002520794	0.039285455
Gm15348	-2.146719593	-1.102133757	0.002523124	0.039285455
Taf6	-1.394479071	-0.479726282	0.002533579	0.039364144
Ccng1	-1.352469369	-0.43559592	0.002533765	0.039364144
Stk36	-1.697822591	-0.763685716	0.002538598	0.039376305
4933439C10I	-1.66113007	-0.732165044	0.002541129	0.039376305
Map3k1	1.637819196	0.711776102	0.002542931	0.039376305
Itih5	-1.504305337	-0.589097429	0.002547447	0.039402934
Hsd11b1	-1.495648891	-0.580771537	0.002555697	0.039487201
Slc25a51	1.392604498	0.477785588	0.002565579	0.039563822
Arf6	1.32153259	0.402212003	0.002568496	0.039563822
Rps28	1.444981335	0.531050858	0.002569079	0.039563822
Crls1	1.289121419	0.366388154	0.002579533	0.039670213
Mccc1os	-3.733951068	-1.900703022	0.002581618	0.039670213
Wnk2	-1.893875633	-0.921341595	0.002590293	0.039760156
Pros1	1.486816484	0.572226589	0.002602217	0.039899721
Pxmp2	-1.421123392	-0.507031825	0.002616198	0.040055251
Ssr2	1.357202643	0.440636145	0.002618046	0.040055251
Hypk	1.31241012	0.392218623	0.002621367	0.040062555
Gabra4	-5.872136917	-2.553885607	0.002627281	0.040109448
Cd8a	-2.978260307	-1.574469854	0.002636641	0.040154479
Il1rl1	3.091254136	1.628192265	0.00263861	0.040154479
Usp20	-1.518910723	-0.603037075	0.00263878	0.040154479
Atmin	-1.34706576	-0.429820281	0.002653879	0.040340671
Tirap	1.752259079	0.809216099	0.002664823	0.04046338
2010107E04I	1.350156437	0.433126576	0.002669145	0.040467832
Lgals3	2.578924715	1.366769658	0.00267086	0.040467832
Stx12	1.390819039	0.475934722	0.002681372	0.040583468
Edem2	-1.368907577	-0.453025045	0.002689512	0.040663003
Ndufb5	1.313898309	0.39385362	0.002695525	0.040710223
Atp5j	1.292261666	0.369898226	0.002700241	0.040711448
Sik1	2.521081551	1.334042787	0.002701384	0.040711448
Nupl1	1.470422102	0.556230357	0.0027043	0.040711848
Rps3a1	1.438933369	0.524999788	0.002708221	0.040727363
Pdhb	1.316676993	0.396901467	0.002717047	0.040816533
Mmp11	-2.125202533	-1.087600337	0.002724425	0.040883778
Tyw3	-2.06716629	-1.047654449	0.002730979	0.040938545
Spopl	1.323626472	0.40449605	0.002740586	0.041038894
Slc20a1	3.170300212	1.664619463	0.002748182	0.041108953
Neu2	-2.463379691	-1.300639014	0.002759698	0.041237444
Sar1a	1.292645832	0.37032705	0.002764133	0.041252704
Eif4e	1.377639677	0.462198599	0.002766575	0.041252704
Klrb1b	2.47806508	1.309214077	0.002770114	0.041261817
Slc44a4	-1.318934839	-0.399373291	0.002773236	0.041264691
Ccbl2	-1.508860345	-0.593459281	0.002783075	0.041367422
Tgm1	5.476697014	2.453306069	0.002801868	0.041602863
Cxx1b	1.433270438	0.519310851	0.002810765	0.041691047
Gja1	2.584428924	1.369845526	0.002837863	0.041996413
3010026O09I	-2.580259135	-1.367515963	0.002840175	0.041996413
Mmrn1	1.623800072	0.699374014	0.002840294	0.041996413
Ept1	1.584116176	0.663678144	0.002844774	0.042018562
4833411C07I	-4.650278939	-2.217317256	0.002853295	0.042095852
Nup62	1.578451777	0.658510186	0.002857718	0.042095852
Fancd2os	-4.181460662	-2.064006991	0.002860081	0.042095852
Ugcg	3.210211465	1.682668334	0.002861956	0.042095852
Iqsec1	-1.502656046	-0.587514818	0.002880095	0.042278471
Dusp18	-1.703612338	-0.768597083	0.002884723	0.042278471
Mrpl33	1.406761843	0.492378109	0.00288484	0.042278471
Hip1r	1.561472933	0.642907562	0.002886374	0.042278471
Esrrb	-1.738932076	-0.798201581	0.002893813	0.042343431
Shc1	1.570706395	0.65141353	0.002901829	0.042416676
Fgd1	-1.62156751	-0.697389088	0.002915206	0.042539464
4930579F01I	-2.88242396	-1.527282549	0.002916267	0.042539464
Pde12	1.614537845	0.691121258	0.002938948	0.042819925
Asl	-1.440907537	-0.526977761	0.002941572	0.042819925
Cacnb3	-1.705143458	-0.769893122	0.002945721	0.042836067
Steap1	2.107250633	1.075361916	0.002950806	0.042865785
2810013P06I	-1.50805212	-0.59268629	0.002977436	0.043170938
Uqcr11	1.332798858	0.414459069	0.002984037	0.043170938
Clic5	-1.352254012	-0.435366178	0.002986342	0.043170938
1810026B05I	1.467530407	0.553390396	0.002986383	0.043170938

BC029722	1.418588388	0.504456043	0.002991268	0.043170938
Acsm2	-1.405999649	-0.491596234	0.002992164	0.043170938
Hn1	2.001673355	1.001206566	0.002993259	0.043170938
Tlr9	-2.822781583	-1.497117502	0.003004331	0.043267589
Rps13	1.395961177	0.481258819	0.003006101	0.043267589
Emx1	-2.22716371	-1.155207609	0.003014799	0.043334898
Ifit2	-3.358848533	-1.747966739	0.003016928	0.043334898
Chka	2.652055002	1.407110696	0.003044616	0.043656121
Aspg	-2.040580498	-1.028979624	0.003045488	0.043656121
Lsm10	-1.438043411	-0.524107228	0.003053337	0.04372416
Tnfaip8	-1.827680063	-0.870013547	0.003058806	0.043758005
Ogfod2	-1.3139466	-0.393906644	0.003068054	0.043826774
Cox17	1.327872124	0.40911622	0.003069834	0.043826774
Slc7a13	-1.887981545	-0.916844662	0.003082092	0.043957248
Adm2	-4.79631821	-2.261927377	0.003090788	0.044036696
Ackr3	1.659131772	0.730428473	0.003097522	0.044065306
Trib1	2.527946249	1.337965788	0.00309905	0.044065306
Klhl28	1.694891209	0.761192674	0.003109309	0.044166604
Asph	-1.339111974	-0.421276601	0.003113735	0.044184937
Mvk	-1.437166256	-0.523226967	0.00312143	0.044228071
1810055G02	1.751728709	0.808779361	0.003123052	0.044228071
Lrfn4	1.814325343	0.859433181	0.003130439	0.044288164
BC021891	-1.776292476	-0.828869148	0.00313699	0.044300873
Rnf145	1.579863375	0.659799801	0.003137625	0.044300873
Wdr11	-1.370833696	-0.45505356	0.003146437	0.04433119
Tbca	1.412493523	0.498244252	0.003148877	0.04433119
Arpc1b	1.759379676	0.815066851	0.00314921	0.04433119
Appl1	1.458661292	0.544644922	0.003154519	0.044360241
Gnb2l1	1.370157266	0.454341495	0.00315757	0.044360241
Optn	1.405546212	0.491130888	0.003175142	0.044530974
Ndufs6	1.287069195	0.364089617	0.003180992	0.044530974
Cdh1	1.987841204	0.991202514	0.003181703	0.044530974
Prune	1.497131333	0.582200784	0.003184532	0.044530974
Pvt1	-1.829208664	-0.871219657	0.003185524	0.044530974
Car9	-2.462488866	-1.300117202	0.003192046	0.044577913
Bola3	1.315336858	0.395432321	0.003198893	0.044629311
Ank2	-1.738279153	-0.797659786	0.003213812	0.044793101
Rhox6	-2.215473079	-1.147614796	0.003220255	0.044819201
Vmac	-1.693170762	-0.759727481	0.003228234	0.044819201
Suv420h2	-1.400601716	-0.486046761	0.003228303	0.044819201
Golm1	1.52098392	0.605004901	0.003228407	0.044819201
Rpl4	1.360771133	0.444424441	0.003237401	0.044899816
Chst7	-1.745315941	-0.80348822	0.003249258	0.045019952
Chil3	8.391841342	3.068987402	0.003271568	0.045284538
Rbm10	-1.404316462	-0.489868083	0.003283392	0.045403614
Inpp5j	-1.632450428	-0.707039183	0.00328826	0.045426343
Wdr83os	1.310953077	0.390616048	0.00329932	0.045520052
Rplp1	1.40536393	0.490943776	0.00330387	0.045520052
Klk1b21	2.215132634	1.147393084	0.00330501	0.045520052
Cops5	1.313214643	0.393102742	0.003307965	0.045520052
Perm1	-3.36864057	-1.752166503	0.003323375	0.045632162
Slc6a19os	7.971628722	2.994874518	0.003323558	0.045632162
Lin7c	1.315044511	0.395111632	0.003328615	0.045632162
Apod	2.61746105	1.388168069	0.003329066	0.045632162
C4a	-3.03022256	-1.599423759	0.003333415	0.04564738
Tnfaip1	1.725044828	0.786633853	0.003337583	0.045660079
Npr2	-1.531515711	-0.614960167	0.003357305	0.045885333
Med29	1.318664215	0.399077244	0.003372029	0.045946498
Bmpr1a	1.324214592	0.405136933	0.003373606	0.045946498
Ddx3y	1.477773916	0.563425569	0.003374707	0.045946498
Ssbp3	-1.713611086	-0.777039718	0.003374823	0.045946498
Shb	1.832634218	0.873918862	0.003404524	0.046306122
Smad1	2.019177626	1.01376783	0.003412591	0.046371096
Hsd17b7	1.338441424	0.420554001	0.003436858	0.04660846
Rps6	1.381318041	0.466045531	0.00344256	0.04660846
Phyhipl	-1.379028554	-0.463652329	0.00344343	0.04660846
Stra6l	1.982704961	0.987470012	0.003447326	0.04660846
Ccdc58	1.352010392	0.435106241	0.003447717	0.04660846
Rsl1d1	1.388779011	0.473817049	0.003449906	0.04660846
Pctp	-1.899121488	-0.925332199	0.003472407	0.046843072
Gadd45a	2.018400685	1.013212601	0.00347392	0.046843072
Adck3	-1.719090258	-0.781645293	0.003480648	0.046888921
Kng2	1.303362086	0.382237934	0.003496258	0.047054218
Zfand5	1.867352113	0.900993992	0.003509429	0.047186413
Cnot6	1.363205224	0.447002769	0.003521895	0.047308889

Rhbdf2	2.505230616	1.324943415	0.003525274	0.047309176
Osbpl1a	-1.359747931	-0.443339231	0.003535201	0.047323754
Cldn7	1.766335423	0.820759333	0.003538227	0.047323754
Amz2	-1.341765238	-0.424132272	0.003539357	0.047323754
Trf	2.211551629	1.145058923	0.0035407	0.047323754
Pou3f3	-1.471027678	-0.556824392	0.003543152	0.047323754
Fam84b	1.644251763	0.717431217	0.003553392	0.047377841
Crocc	-3.00236209	-1.586097979	0.003553926	0.047377841
Syncrip	1.484047999	0.569537755	0.003577135	0.047571147
Mafk	1.73730231	0.796848821	0.003577596	0.047571147
Ptbp3	1.590866631	0.669812893	0.003580571	0.047571147
Tln2	-2.078907925	-1.055825863	0.003584253	0.047571147
Hk1	-1.328840032	-0.410167441	0.003586927	0.047571147
LOC1026317	-2.393453225	-1.259093612	0.003588683	0.047571147
Snx25	-1.301719204	-0.380418276	0.003592326	0.047574688
2010107G23	-1.519759387	-0.60384293	0.00360997	0.047748055
Cyp4b1	-1.345581818	-0.428230117	0.003612194	0.047748055
Lppos	-1.78927025	-0.839371308	0.003637372	0.048035805
Sun3	-1.82410187	-0.867186302	0.003664062	0.048340004
Rhoa	1.398741877	0.484129753	0.003667267	0.048340004
Lztr1	-1.40277118	-0.488279696	0.00368483	0.048521719
Vps8	-1.442574254	-0.528645581	0.00368794	0.048521719
Rpl15	1.314532607	0.394549928	0.003695203	0.048571924
Zfp133-ps	-3.215291454	-1.684949518	0.003703605	0.048626268
Tarsl2	-1.490856455	-0.576141356	0.003707175	0.048626268
Dnajc5	1.466133159	0.55201614	0.00370969	0.048626268
Rcn2	1.353530393	0.436727283	0.00372277	0.048653327
Ces1e	-1.536304526	-0.619464215	0.003725115	0.048653327
Raf1	1.409404249	0.495085469	0.003725898	0.048653327
Rnd1	3.311125741	1.727321799	0.003729251	0.048653327
Rhot2	-1.478784928	-0.564412245	0.003731514	0.048653327
9530082P21	-1.820680287	-0.864477606	0.003732809	0.048653327
Arpc3	1.421404576	0.507317249	0.003735924	0.048653327
Usp53	1.759069312	0.81481233	0.00375118	0.04880691
Wbscr27	-1.491391389	-0.576658917	0.003758169	0.048852735
Ahcy	-1.284241663	-0.360916708	0.003764893	0.048869728
Pbld1	-2.225674198	-1.154242421	0.003766413	0.048869728
Kcnd3	1.827841697	0.870141129	0.003771602	0.048892032
Mapre2	-1.324390049	-0.405328076	0.003790592	0.049093045
Dnm3	-1.543721069	-0.626412099	0.003800522	0.049165559
Appl2	-1.48261187	-0.568140967	0.003805913	0.049165559
Chmp2a	1.399315321	0.484721095	0.003806658	0.049165559
Tmem120a	2.300334783	1.201843841	0.003815803	0.049197414
Per3	-2.050433196	-1.035928741	0.003816108	0.049197414
Stx3	1.623169528	0.698813687	0.003852684	0.049514735
Pgpep1l	-2.253795373	-1.172356536	0.003855995	0.049514735
Ccpg1os	-1.510199322	-0.594738975	0.003859716	0.049514735
Dcaf8	-1.348139147	-0.430969411	0.003862442	0.049514735
Cycs	1.403190773	0.488711166	0.003863843	0.049514735
Trim37	-1.490199853	-0.575505826	0.003865047	0.049514735
Map2k3	1.560790487	0.642276889	0.003870453	0.049514735
Apba3	-1.311227665	-0.390918199	0.003872809	0.049514735
Usmg5	1.330464251	0.411929746	0.003875002	0.049514735
Ppapdc1b	1.580341764	0.660236588	0.003875861	0.049514735
Cmbl	-1.401795673	-0.487276077	0.003880899	0.049534193
Atrnl1	-1.312068632	-0.391843187	0.003887451	0.04957291
Iqsec2	-1.653746004	-0.72573767	0.003892706	0.049576353
March10	-3.453198504	-1.787933269	0.003896624	0.049576353
Ppp2cb	1.566126663	0.647200897	0.003898275	0.049576353
Csrnp1	2.803627696	1.487294781	0.003906924	0.049638873
Pik3r1	1.726209175	0.787607295	0.00391099	0.049638873
Katnbl1	1.368262526	0.452345064	0.003915698	0.049638873
Hbegf	3.389073342	1.760890858	0.003917282	0.049638873
Prkcq	-1.771916949	-0.825310985	0.003931293	0.049771654
Misp	-1.547004667	-0.629477549	0.003941186	0.049852108
Ifi27	1.407137115	0.492762916	0.00395205	0.0499168
Cep97	-1.600144162	-0.678201888	0.003953385	0.0499168

GeneSymbo	HFD_IRI_vs_ND_IRI_FC	HFD_IRI_vs_ND_IRI_logFC	HFD_IRI_vs_ND_IRI_PValue	HFD_IRI_vs_ND_IRI_FDR
**Acsl4**	2.632643596	1.396512224	1.0499E-06	0.000219422
Dhcr7	-1.779916156	-0.831809284	0.002258345	0.036750285
Slc40a1	1.479435786	0.565047078	0.005028707	0.055902044
Lpcat3	1.212626905	0.278135737	0.05404588	0.200621836
**Ptgs2**	1.794977118	0.843965453	0.064843899	0.221951087
Hmox1	-1.352907604	-0.436063314	0.546254732	0.737852322
Keap1	-1.040514239	-0.057296708	0.667217009	0.818682939
Gch1	5.140297787	2.36185194	0.002103247	0.035321195
Nfe2l2	1.562930363	0.6442535	0.002397384	0.037999472
Msmo1	1.800312177	0.848247094	0.018626361	0.110140181
Slc7a11	6.672326768	2.738189945	0.021657	0.120477215
Sc5d	-1.163769848	-0.218805773	0.301015192	0.531865164
Ebp	1.079332371	0.110139199	0.523369067	0.720041725
